# Surface and subsurface oceanographic features drive forage fish distributions and aggregations: Implications for prey availability to top predators in the US Northeast Shelf ecosystem

**DOI:** 10.1002/ece3.10226

**Published:** 2023-07-11

**Authors:** Chandra Goetsch, Julia Gulka, Kevin D. Friedland, Arliss J. Winship, Jeff Clerc, Andrew Gilbert, Holly F. Goyert, Iain J. Stenhouse, Kathryn A. Williams, Julia R. Willmott, Melinda L. Rekdahl, Howard C. Rosenbaum, Evan M. Adams

**Affiliations:** ^1^ Biodiversity Research Institute Portland Maine USA; ^2^ Northeast Fisheries Science Center Narragansett Rhode Island USA; ^3^ CSS, Inc. Fairfax Virginia USA; ^4^ National Centers for Coastal Ocean Science NOAA Silver Spring Maryland USA; ^5^ Normandeau Associates Gainesville Florida USA; ^6^ Wildlife Conservation Society, Ocean Giants Program, Bronx Zoo Bronx New York USA

**Keywords:** forage fish aggregation, hierarchical Bayesian model, joint species distribution model, predator–prey interactions, trophodynamics

## Abstract

Forage fishes are a critical food web link in marine ecosystems, aggregating in a hierarchical patch structure over multiple spatial and temporal scales. Surface‐level forage fish aggregations (FFAs) represent a concentrated source of prey available to surface‐ and shallow‐foraging marine predators. Existing survey and analysis methods are often imperfect for studying forage fishes at scales appropriate to foraging predators, making it difficult to quantify predator–prey interactions. In many cases, general distributions of forage fish species are known; however, these may not represent surface‐level prey availability to predators. Likewise, we lack an understanding of the oceanographic drivers of spatial patterns of prey aggregation and availability or forage fish community patterns. Specifically, we applied Bayesian joint species distribution models to bottom trawl survey data to assess species‐ and community‐level forage fish distribution patterns across the US Northeast Continental Shelf (NES) ecosystem. Aerial digital surveys gathered data on surface FFAs at two project sites within the NES, which we used in a spatially explicit hierarchical Bayesian model to estimate the abundance and size of surface FFAs. We used these models to examine the oceanographic drivers of forage fish distributions and aggregations. Our results suggest that, in the NES, regions of high community species richness are spatially consistent with regions of high surface FFA abundance. Bathymetric depth drove both patterns, while subsurface features, such as mixed layer depth, primarily influenced aggregation behavior and surface features, such as sea surface temperature, sub‐mesoscale eddies, and fronts influenced forage fish diversity. In combination, these models help quantify the availability of forage fishes to marine predators and represent a novel application of spatial models to aerial digital survey data.

## Graphical abstract

Forage fishes are a critical food web link in marine ecosystems, forming surface‐level forage fish aggregations (FFAs) that represent a concentrated source of available prey for surface‐ and shallow‐foraging marine predators. We used hierarchical Bayesian models to examine the oceanographic drivers of forage fish community distributions from bottom trawl data and surface FFAs from aerial digital survey data in the US Northeast Continental Shelf ecosystem. Our results indicate that while bathymetric depth contributed to both distribution patterns, subsurface oceanographic features primarily influenced aggregation behavior and surface features were more influential to forage fish community patterns.

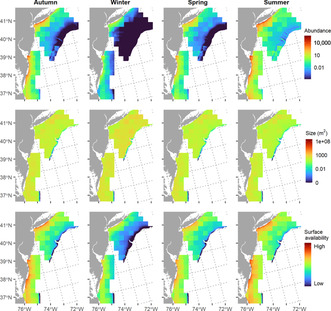

## 1 INTRODUCTION

Prey availability, a function of the density of prey resources and their accessibility to predators, is an important factor affecting the abundance and distribution of marine species (Frederiksen et al., 2006). Marine prey species are hierarchically organized over multiple spatial and temporal scales with individuals grouping to form cohesive aggregations (e.g., swarms, schools, or shoals) at fine scales (<1 km) and aggregations forming distinct organizational patterns at submesoscales (1–10 km) and mesoscales (10–1000 km) across the broader regional seascape (spatial extents >10,000 km^2^; Fauchald et al., 2000; Russell et al., 1992; Steele, 1978). Frequently, marine prey distributions are described at coarser mesoscale resolutions, simplified as general occupancy (i.e., presence or absence) and/or integrated over the water column (Arkema et al., 2006; Ruckelshaus et al., 2008). These generalizations discount the patchy nature of prey availability at smaller scales, which many marine predators target within the broader prey distribution to increase foraging efficiency and success (Fauchald et al., 2000; Wellenreuther & Connell, 2002). Thus, while the broad‐scale distribution of prey may set the limits of marine predator distribution, the timing and spatial patterns of prey aggregations determine realized prey availability, impacting the fine and submesoscale habitat use of predators.

Small, schooling pelagic forage fishes are a critical prey resource within marine food webs, linking primary production and zooplankton to upper trophic level predators, such as seabirds, seals, cetaceans, piscivorous fishes, and squids (Cury et al., 2000; Pikitch et al., 2012). Forage fishes form large, dense aggregations in a hierarchical patch structure that varies over fine spatial and temporal scales (Freon & Misund, 1999; Pitcher, 1986). Although other groups such as squids and juvenile stages of some piscivorous fishes (e.g., age 0–1 groundfish), also exhibit schooling behavior and can serve a similar functional role, small pelagic forage fishes remain in this role throughout their life history and are the primary forage species in many marine ecosystems (Rountos, 2016). The formation and distribution of forage fish aggregations (FFAs) are driven by a combination of their responses to the physical abiotic environment (e.g., physiological thermal constraints) and responses based on biotic interactions (e.g., foraging, predator avoidance, and spawning; Genin, 2004; Pitcher, 1986). Surface‐level FFAs, in particular, are important for surface or shallow‐foraging predators (e.g., plunge‐diving or dipping seabirds; Fauchald, 2009) and predators that trap aggregated forage fishes between themselves and the surface as a foraging strategy (e.g., cetaceans, sharks, and pursuit‐diving seabirds), which often form multispecies feeding associations for efficient exploitation (Thiebault et al., 2016).

Differences in survey and sampling methods between forage fishes and their predators make it challenging to obtain prey availability data at behaviorally relevant scales to discern predator–prey relationships, resulting in a fundamental scale mismatch between predator and prey data (Benoit‐Bird et al., 2013; Fauchald et al., 2000). For instance, bottom trawl surveys are routinely used in fisheries stock assessments to discern abundance and distribution of multiple fish species across broad seascape areas (Despres‐Patanjo et al., 1988). Bottom trawl surveys are not optimal for sampling low to mid‐trophic level pelagic forage fishes, since the gear has species‐ and size‐dependent selectivity, and in deeper waters may only reliably sample pelagic, schooling forage fishes upon deployment and recovery as the net moves vertically through the water column. Nonetheless, while forage fishes are primarily mid‐water species, they do use the full water column over the continental shelf via several mechanisms (i.e., diel vertical migration, predator avoidance, spawning, and over‐wintering; Freon & Misund, 1999). In the Northeast U.S. Continental Shelf ecosystem (NES), forage fishes are routinely captured in bottom trawls and the distribution of these captures is systematic, likely representing true broadscale distribution tendencies (Friedland et al., 2023; Roberts et al., 2022; Suca, Deroba, et al., 2021). However, bottom trawl surveys are ill‐suited for monitoring the distribution of surface‐level FFAs on which many predators rely. Large‐scale active acoustic surveys are widely used for conducting abundance (i.e., biomass) surveys of forage fishes (Jech & McQuinn, 2016; McQuinn, 2009). Yet, acoustic surveys for assessing FFA characteristics specifically, including horizontal and vertical distribution, school density, and predator–prey interactions (Lucca & Warren, 2019; Thayne et al., 2019) are often conducted at small spatial scopes, and seascape‐level patterns in the distributions of FFAs are largely unknown.

Despite the importance of comparing predator foraging success and behavior to prey distributions at interaction‐level scales (Fauchald, 2009; Russell et al., 1992), forage fishes are frequently compared with predators at much coarser scales or omitted from analyses of predator distributions entirely due to data paucity, resulting in, at best, unexplained variance in trophic responses or, at worst, an inability to detect meaningful trophic relationships (Hunt & Schneider, 1987; Levin, 1992). These challenges have prevented clear tests of predator–prey hypotheses at relevant scales, and many studies have not found strong relationships between forage fish distributions and marine predators (Fauchald, 2009; Grémillet et al., 2008; Russell et al., 1992; Torres et al., 2008).

Forage fishes are often characterized by asynchronous “boom and bust” population cycles, resulting in high temporal variability in species dominance (Schwartzlose et al., 1999). In addition, forage fishes form large, multispecies aggregations, which vary spatiotemporally across scales (Cury et al., 2000; Engelhard et al., 2014). Consequently, many marine predators are not dependent on a single forage fish species, instead favoring generalist feeding strategies or engaging in prey‐switching behavior (Cury et al., 2000). In fact, local density, species composition, and spatial availability of forage fishes generally are key factors in the foraging and reproductive success of marine predators (Benoit‐Bird et al., 2013; Davoren, 2013). Community‐level measures of forage fish distribution or abundance may be an indicator of realized prey availability for opportunistic generalist predators (Koehn et al., 2016). Understanding forage fish community dynamics and the oceanographic features driving these patterns may be more applicable to predator–prey studies than examining these patterns at the individual prey species level. Advances in joint species distribution modeling (JSDM) allow for the estimation of community‐level distributions, environmental niches, and species associations (Ovaskainen & Abrego, 2020; Ovaskainen et al., 2017; Roberts et al., 2022; Warton et al., 2015), which may be more relevant in some cases to marine predator–prey relationships than the results of single‐species habitat modeling.

Aircraft‐based aerial digital video/photographic surveys, designed to target seabirds, marine mammals, and other marine predators, are a technological advancement that enables the reliable detection of surface FFAs across regional seascapes (Buckland et al., 2012; Taylor et al., 2014). Observations of surface FFAs from digital aerial surveys allow for investigation of processes driving realized prey availability, as FFAs integrate both interaction‐level scales (i.e., prey patch distribution) and community‐level dynamics (i.e., aggregations can represent multiple species). In the absence of FFA data, many studies of marine predator distributions rely on oceanographic features, such as bathymetry, sea surface temperature (SST), and chlorophyll concentration, as proxies for prey availability (Becker et al., 2016; Palacios et al., 2014; Torres et al., 2008), assuming these features adequately represent prey patterns. For example, some seabird species have been associated with frontal features, which have been interpreted as an aggregating mechanism for prey (Scales, Miller, Embling, et al., 2014). While some of these oceanographic features are known to play a role in the general distribution of forage fish species (Friedland et al., 2019; Suca, Deroba, et al., 2021), we lack an understanding of the physical and biological mechanisms driving the formation and distribution of surface FFAs (Cox et al., 2018; Peck et al., 2021). In addition, data on relevant oceanographic variables, such as subsurface features, are often lacking at appropriate spatiotemporal scales for modeling dynamic, ephemeral processes such as aggregation formation, leading to an incomplete understanding of the oceanographic processes driving forage fish distribution and aggregation (Brodie et al., 2018; Mannocci et al., 2017).

Data from aerial digital surveys offer a novel opportunity to assess the oceanographic processes driving the abundance and size of FFAs and whether those differ from the drivers of forage fish occurrence distributions. Surface FFAs are a product of forage fish presence and a behavioral response. Thus, distributions of FFAs are not necessarily driven by the same environmental features as broadscale occurrence. Static habitat features (i.e., bathymetric depth and bottom topography) can interact with dynamic ocean processes, creating conditions that promote the formation of FFAs (Genin, 2004; Holland et al., 2021). For example, zooplankton in coastal waters can accumulate via currents and high chlorophyll‐*a* along productivity fronts; shallow topography then prevents downward migration of zooplankton, driving increased surface FFAs which forage on the concentrated plankton (Holland et al., 2021). Subsurface dynamic processes, such as stratification, also influence aggregating mechanisms via concentrating nutrients, subsurface productivity, and zooplankton (Genin, 2004). Abrupt changes in bottom topography can interact with water column stratification to drive FFAs to the surface (Cox et al., 2018).

We used models of the forage fish community distribution alongside independent models of FFA distribution to address the following questions:

1. Do broadscale distributions of the forage fish community in the NES ecosystem, as determined from long‐term bottom trawl surveys, demonstrate relationships to patterns of surface prey availability, as determined from aerial digital surveys of surface FFAs?
2. Which oceanographic processes are driving the spatial distributions of forage fishes at these differing organizational scales (broadscale occupancy distribution vs. surface FFA distribution)?
3. Where are regions of high realized prey availability?

To examine broad patterns in forage fish community dynamics, we modeled the joint distribution of 15 surface‐aggregating forage fish species from the NES in autumn and spring. We used digital aerial survey data of surface FFAs from the New York and Mid‐Atlantic Bights to model the spatial distribution of FFA abundance and size by season. To determine which oceanographic processes influence prey availability and broad‐scale forage fish distribution, we included a set of environmental covariates representing three categories: static physical habitat (i.e., bathymetric measures), dynamic surface processes (i.e., temperature and chlorophyll fronts, eddies), and dynamic subsurface processes (i.e., stratification). We predicted that different environmental processes would drive forage fish distributions and surface FFAs with dynamic features more influential to FFAs. We found that multiple models describing forage fish distributions at community and aggregation levels can provide a more complete picture of the conditions driving the broadscale distribution and aggregation behavior of forage fishes, and, thus, prey availability for surface‐ and shallow‐foraging marine predators.

## 2 MATERIALS AND METHODS

### 2.1 Study region

**FIGURE 1:**
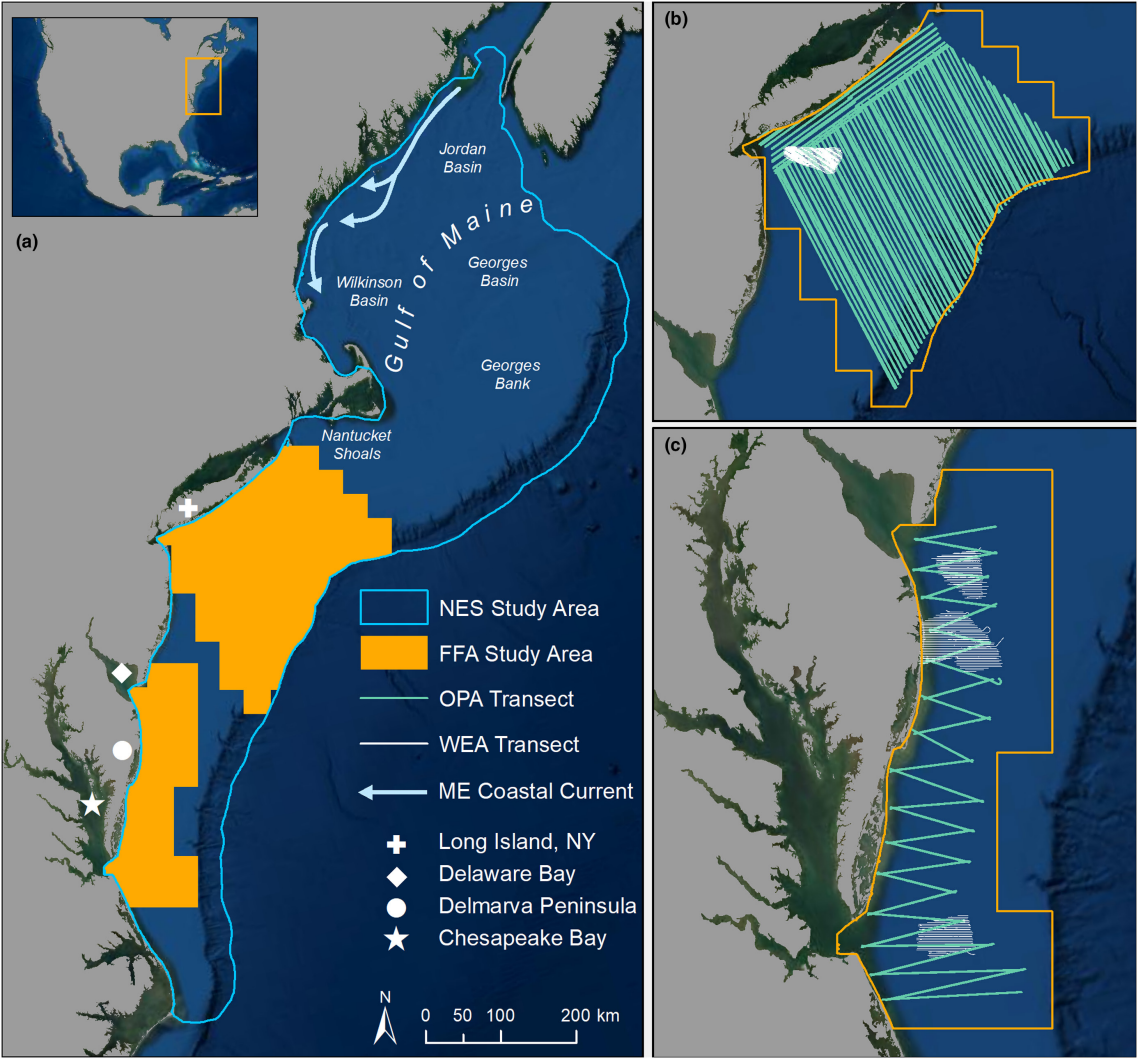
(a) US Northeast Continental Shelf (NES) study area, (b) New York (NY) Bight aerial digital survey transects, and (c) Mid‐Atlantic Bight aerial digital survey transects. Model prediction extents are depicted via blue (NES) and orange (FFA) outlines. Relevant geographic features are labeled (see legend). FFA, forage fish aggregation; ME, Maine; OPA, offshore planning area; WEA, wind energy area.

This study has multiple nested regions, the largest being the NES, a well‐studied marine ecosystem (Sherman & Skjoldal, 2002), encompassing the shelf waters along the western boundary of the North Atlantic Ocean from Cape Hatteras, North Carolina to the Gulf of Maine (Figure 1a). Within the NES, two study areas, the New York Bight (Figure 1b; Robinson Willmott et al., 2021) and the Mid‐Atlantic Bight (Figure 1c; Williams et al., 2015) were aerially surveyed with high‐resolution digital cameras to estimate spatial distributions of marine animals.

### 2.2 Data description

#### 2.2.1 Bottom trawl surveys—forage fish species/community data

The NOAA Northeast Fisheries Science Center has conducted a biannual fisheries‐independent bottom trawl survey across the NES ecosystem for over 50 years (1968–2019; Grosslein, 1968, Appendix 1: Section 1). Bottom trawl surveys are conducted in the boreal autumn and spring, employing a random stratified survey design with strata based primarily on depth and secondarily on latitude (Despres‐Patanjo et al., 1988). Within strata, tow locations are assigned randomly prior to each seasonal survey. A minimum of two locations are sampled per strata, totaling ~300 locations per season. The trawl net has a 12.5 mm mesh liner at the codend to retain juvenile and small‐bodied fishes. We used tow data with catch identification at the species level for 15 pelagic, schooling forage fishes (Table 1). Data were standardized using calibration factors to account for vessel and gear changes in the surveys during the time series (Miller et al., 2010). However, the bottom trawl gear was not designed to capture pelagic forage fishes; thus, even after standardization for temporal gear changes, the abundance/biomass data may not be fully representative of forage fishes. Therefore, we transformed raw abundance/biomass per tow to binary occupancy data (presence/absence) for distribution modeling.

**TABLE 1:** Occurrence (no. of tows present) of surface schooling forage fishes from the bottom trawl surveys of the US Northeast Continental Shelf (NES) study area included in the community distribution models.

Code	Common name	Scientific name	Family	Occurrences^a^	
				Autumn	Spring
alewif	Alewife	*Alosa pseudoharengus*	Clupeidae	**1124**	**3336**
atherr	Atlantic thread herring	*Opisthonema oglinum*	Clupeidae	**337**	1
atlher	Atlantic herring	*Clupea harengus*	Clupeidae	**2082**	**3738**
atlmac	Atlantic mackerel	*Scomber scombrus*	Scombridae	**816**	**2033**
atlmen	Atlantic menhaden	*Brevoortia tyrannus*	Clupeidae	**115**	**99**
atlsil	Atlantic silverside	*Menidia menidia*	Atherinopsidae	12	**536**
atsaur	Atlantic saury	*Scomberesox saurus*	Scomberesocidae	**191**	1
bayanc	Bay anchovy	*Anchoa mitchilli*	Engraulidae	**660**	**231**
bluher	Blueback herring	*Alosa aestivalis*	Clupeidae	**496**	**2007**
butter	Atlantic butterfish	*Peprilus triacanthus*	Stromateidae	**4728**	**1806**
rherri	Round herring	*Etrumeus teres*	Dussumieriidae	**773**	17
sandal	Northern sand lance	*Ammodytes dubius*	Ammodytidae	**327**	**703**
silanc	Silver anchovy	*Engraulis eurystole*	Engraulidae	**153**	0
spsard	Spanish sardine	*Sardinella aurita*	Clupeidae	**197**	0
stranc	Striped anchovy	*Anchoa hepsetus*	Engraulidae	**692**	**21**

^a^ Bold font indicates the species had >20 occurrences and was included in the model for that season.

#### 2.2.2 Aerial digital surveys—forage fish aggregation data

High‐resolution aerial digital surveys were conducted as baseline ecological studies of designated offshore planning areas (OPAs; Figure 1b,c) for wind energy development and were designed to estimate patterns of above‐water and surface‐level fauna. Detectability of submerged FFAs in these surveys varies due to water turbidity and weather conditions with the estimated average vertical penetration of the water column being ~3 m and a maximum penetration under ideal conditions of ~8–9 m (Hodgson et al., 2017; Martin Scott, HiDef Aerial Surveying, Ltd., pers. comm). Therefore, this data, including subsequent analysis and interpretation, represents only surface FFAs. Observations of the number and size of surface FFAs were collected from two aerial digital survey projects (Appendix 1: Section 1): (1) the New York State Energy Research and Development Authority (NYSERDA) Digital Aerial Baseline Survey of Marine Wildlife in Support of Offshore Wind Energy project (hereafter, New York project) and (2) Department of Energy (DOE) Mid‐Atlantic Baseline Studies project (hereafter, Mid‐Atlantic project). The New York project conducted aerial transect surveys (*n* = 12) over the New York Bight (43,745 km^2^, Figure 1b) quarterly over 3 years (2016–2019; Appendix 1: Section 1). High‐resolution images were collected using two still camera systems (Shearwater II and III), both with a 1.5 cm ground sampling distance (GSD; Robinson Willmott et al., 2021). The New York project surveyed the OPA with 584 m wide linear transect strips for 7% coverage in all 3 years. In 2016, a higher‐resolution grid survey (330 × 219 m) with 10% coverage was also conducted across the smaller wind energy area (WEA). The Mid‐Atlantic project conducted aerial transect surveys (*n* = 15) over the Mid‐Atlantic Bight (13,245 km^2^; Figure 1c) from March 2012 to May 2014 (Appendix 1: Section 1) with four belly‐mounted high‐resolution video cameras (Gen II), creating 200 m wide transect strips. Initial surveys (*n* = 3) in 2012 used a combination of 2 and 3 cm GSD, adjusted to only 2 cm GSD for the remainder of the study to increase image clarity and color rendition for improved species identification across all taxa (Hatch et al., 2013). High‐density parallel transect surveys (1 km spacing) were conducted in each of the smaller WEAs, providing ~20% coverage, while the remainder of the OPA was surveyed via a sawtooth transect path with ~2% coverage.

For both projects, FFAs were identified from the transect image data using detection software and manual review methods followed by quality control (Buckland et al., 2012; Duron et al., 2015; Hatch et al., 2013; Normandeau Associates Inc., 2020). FFAs were identified as cohesive groups of similarly sized individuals with synchronous swimming behavior, where individuals within the group were indistinguishable due to group density and small body size. Species composition of FFAs was not identifiable due to submersion and small body size, but mackerel, menhaden, herring, and hickory shad are major schooling species in the New York Bight (Normandeau Associates Inc., 2020), while menhaden, mackerel, herring, bay anchovy, alewife, and blueback herring are frequent schoolers in the Mid‐Atlantic (Williams et al., 2015). The vertical height of the FFAs could not be determined from the imagery, so we could not estimate FFA volume. Instead, FFA size was defined as the visible surface area (m^2^) of each FFA, where the entire FFA was manually traced, enhancing the color of the image as necessary to determine the aggregation edges (Streampix 8, Norpix). Calibrations to account for the flight altitude and pixel resolution were applied to estimate the size of each shoal in m^2^ from the digital image.

For the FFA analysis, we aggregated FFA abundance and size data to a 4 × 4 km grid overlaid on the FFA study area (i.e., the combined area of both projects). For FFA abundance, FFAs were summed for each 4 × 4 km grid cell (*n* = 3361) by survey and season. Survey effort per grid cell was calculated by summing the total area of ground surveyed (km^2^) in that grid cell by survey and season. The 4 × 4 km grid was nested within a larger 32 × 32 km grid, which was used in the FFA model to account for spatial autocorrelation in the data (see Section 2.4).

### 2.3 Environmental data

We included a combination of static habitat features and dynamic oceanographic processes in our models as environmental covariates. Initially, we considered 28 environmental covariates (13 static, 14 dynamic), encompassing a range of surface and subsurface features obtained from publicly available oceanographic data sources (Appendix 1: Tables A1 and A2). Static habitat included bathymetric terrain measures (e.g., depth, slope, rugosity) and sediment grain size. Dynamic covariates included remote‐sensed, modeled, and derived data for surface and subsurface features (Appendix 1: Table A2). We calculated the SST seasonal anomaly (hereafter, SST anomaly) by dividing each SST value by the seasonal SST mean across the FFA 4 × 4 km grid (Figure 1a). SST and chlorophyll fronts and frontal metrics (i.e., *Fprob*: front persistence, *Fmean*: front intensity; Table 2) were derived from the raw SST and chlorophyll remote‐sensed data products (see Appendix 1: Section 2 for detailed methods). All covariates were resampled with bilinear interpolation to a 4 × 4 km grid (spatially concurrent with the FFA 4 × 4 km grid) encompassing the NES study area. Dynamic covariates were used at a daily temporal resolution, matching the observation date.

**TABLE 2:** Summary of environmental covariates included in the (1) forage fish community models and (2) forage fish aggregation (FFA) models.

	Covariate	Model	Description	Type
**Static habitat**	BPI	2	Benthic position index: second order derivative of bathymetric depth; negative values = valleys, positive values = peaks, values near 0 indicate flats	Bathymetric
	Depth (log)	1, 2	Log of the seafloor bathymetric elevation	Bathymetric
	Rugosity^a^	1	Variation in the amplitude of the height of the bathymetric terrain as given by the ratio of the actual to the geometric surface area; values near 1 indicate flats (lowest bathymetric complexity) and higher values indicate increasing complexity	Bathymetric
	Sediment	1, 2	Benthic soft sediment grain size (mm)	Bathymetric
**Dynamic processes**	Chl*a*	1, 2	Copernicus GlobColour chlorophyll‐*a* interpolated cloud‐free product	Surface
	Chl *Fprob* ^b^	1, 2	7‐day composite probability of observing a Chl*a* front, indicating front persistence	Surface
	Chl *Fmean* ^b^	1	7‐day composite mean Chl*a* front gradient: front intensity	Surface
	FSLE	1, 2	Finite‐size Lyapunov exponents; identifies Lagrangian coherent structures: submesoscale eddies and filaments (~10 km scale)	Surface
	MLD	1, 2	Mixed layer depth from the GLORYS12V1 global ocean eddy‐resolving model	Subsurface
	Salinity	1, 2	Sea surface salinity from the GLORYS12V1 global ocean eddy‐resolving model	Surface
	SST	1	OSTIA global sea surface temperature gap‐free reprocessed product	Surface
	SST anomaly	2	SST seasonal anomaly: difference from the seasonal SST mean of the study area	Surface
	SST *Fprob* ^b^	1, 2	7‐day composite probability of observing an SST front, indicating front persistence	Surface
	SST *Fmean* ^b^	1	7‐day composite mean SST front gradient: front intensity	Surface

^a^ Rugosity was only included in the autumn community models.

^b^ We ran two alternative community models for both seasons, substituting the two *Fmean* covariates for the *Fprob* versions.

Final covariate sets (see Table 2 for abbreviation definitions and descriptions) were selected for each model after examining pairwise Pearson correlation coefficients (−.6 > *r* > .6) and assessing multi‐collinearity with variance inflation factors (VIF < 3; Zuur et al., 2009). We aimed to use a common covariate set to compare the community distribution and FFA models, but this was not possible due to differences in correlations and multi‐collinearity for the datasets. Covariate substitutions among models were made to represent similar features (e.g., SST anomaly for SST). The final covariate sets represented static habitat, dynamic surface, and dynamic subsurface features (see Section 2.4 and Table 2 for details). All covariates were mean‐centered and variance‐scaled prior to analysis.

### 2.4 Model description and evaluation

#### 2.4.1 Forage fish community models

We applied JSDMs to model the forage fish community in the NES, using the HMSC R‐package (Hierarchical Modeling of Species Communities, version 3.0‐12; Ovaskainen & Abrego, 2020; Tikhonov et al., 2020). HMSC uses a Bayesian multivariate hierarchical generalized linear mixed model framework to simultaneously fit all species distribution models and infer joint interactions among species and environmental covariates (Ovaskainen & Abrego, 2020; Ovaskainen et al., 2017). The HMSC framework uses latent variables to model random effects and estimates residual species associations, providing inference on species co‐occurrences patterns not explained by responses to the environmental covariates (Ovaskainen & Abrego, 2020).

We restricted our analysis to the years 1997–2019 (autumn) and 1998–2019 (spring) due to the availability of remote‐sensed chlorophyll data. We selected species with enough presence detections (hereafter, occurrences) in the data to achieve a well‐fitting model (>20). The HMSC framework leverages the relationships among species to accurately model rare community members (Erickson & Smith, 2023; Ovaskainen & Abrego, 2020). The autumn survey (7305 sampling tows) collected 14 species of schooling forage fish with >20 occurrences: alewife, Atlantic thread herring (hereafter, thread herring), Atlantic herring (hereafter, herring), Atlantic mackerel (hereafter, mackerel), Atlantic menhaden (hereafter, menhaden), Atlantic saury (hereafter, saury), bay anchovy, blueback herring, butterfish, round herring, northern sand lance, silver anchovy, Spanish sardine, and striped anchovy (see Table 1 for scientific names). In contrast, the spring survey (7225 tows) only collected 10 forage fish species with >20 occurrences: alewife, herring, mackerel, menhaden, Atlantic silverside (hereafter, silverside), bay anchovy, blueback herring, butterfish, northern sand lance, and striped anchovy (Table 1).

Briefly, we describe the application of HMSC to the NOAA bottom trawl data (see Ovaskainen & Abrego, 2020 for a complete model description). Independently for autumn and spring, we modeled species occurrence as a function of the environmental covariates with a probit link regression mixed model. To account for residual variation not explained by the covariates, we specified two random effects: (1) a tow‐level random effect to control for unexplained variance at the sampling level and (2) a temporally explicit random effect of year, modeled with an exponentially decaying covariance structure, to account for annual variation in occupancy. The residual species associations are taken from the species‐to‐species variance–covariance matrix derived via the random effects (Ovaskainen & Abrego, 2020). Due to strong correlations between the two frontal metrics and no a priori expectation of predictive strength for *Fmean* versus *Fprob* covariates, we ran two alternative models for both seasons, substituting the two *Fmean* covariates for the *Fprob* versions. The autumn covariate set included depth (log), rugosity, sediment, Chl*a*, Chl *Fprob* (*Fmean*), FSLE, MLD, salinity, SST, and SST *Fprob* (*Fmean*). The spring covariate set was simplified by dropping rugosity to achieve model convergence (Table 2).

**FIGURE A1:**
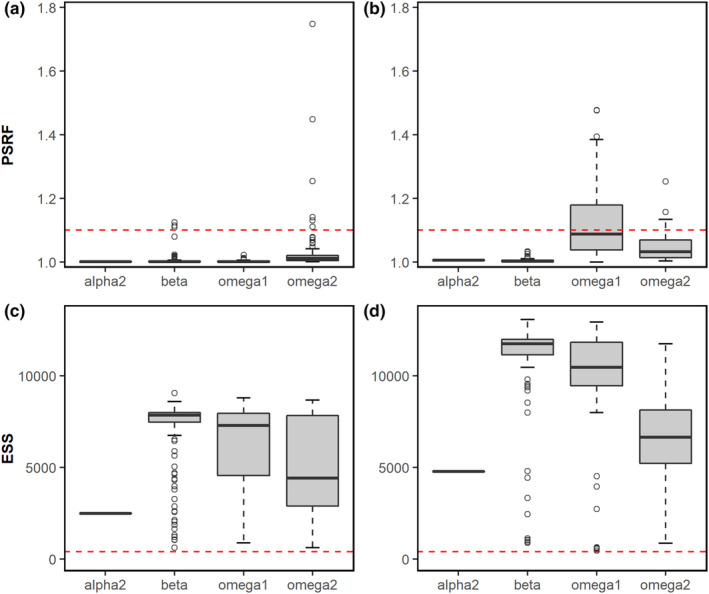
Potential scale reduction factors (PSRFs) and effective sample size (ESS) for monitored parameters to evaluate autumn (a, c) and spring (b, d) HMSC model convergence for the best fitting *Fprob* models. For the PSRF values, the autumn model had 96.6%, and the spring model had 82.4% below 1.1 (red dashed line). The 0.95 quantiles for PSRF were 1.06 and 1.26 for autumn and spring, respectively. The ESS values of all parameters for both models are above 400 (red dashed line).

We fit the models using the default prior distributions (Ovaskainen & Abrego, 2020) and sampled the posterior distribution with four Markov Chain Monte Carlo (MCMC) chains (see Appendix 2: Table A1 for MCMC sampling parameters). MCMC convergence was examined visually and by calculating the potential scale reduction factors (PSRF; i.e., $\hat{R}$) and effective sample size (ESS) of the alpha (spatial scale of the temporal random effect), beta (fixed effects), and omega (species associations per random effect) parameters (Brooks & Gelman, 1998; Gelman & Rubin, 1992). We considered the model adequately converged if the mean and median PSRF values for the alpha (factor 1), beta, and omega parameters were less than 1.1 and if the ESS values were more than 400 (Appendix 2: Figure A1; Vehtari et al., 2021).

To determine the best‐performing model (*Fprob* or *Fmean* version) for autumn and spring, we compared the Watanabe‐Akaike Information Criterion (WAIC) scores (Watanabe, 2013). For the best seasonal models, we evaluated the explanatory power, predictive accuracy, and conditional predictive accuracy by computing the area under the receiver operator curve (AUC; Pearce & Ferrier, 2000). Explanatory power was calculated using the model fitted to all data to get species‐specific AUC values and a summarized mean AUC. For predictive accuracy, we performed a threefold cross‐validation analysis, randomly assigning each year of sampling data to one of the three folds, and computing predictions for each fold (i.e., the testing data) based on the model fitted to the remaining two folds (i.e., the training data). The number of folds chosen was in keeping with best practices for HMSC analysis, while allowing for a reasonable computational time frame. For conditional predictive accuracy, we conducted conditional cross‐validation to evaluate the importance of estimated species associations to model predictions, where the species‐to‐species variance–covariance matrix is employed along with the estimated covariate parameters to make predictions (for details see Ovaskainen & Abrego, 2020). Finally, to assess the contributions of the fixed and random covariates to model fit, we partitioned the species‐specific explained variance between the environmental covariates and each of the random effects.

#### 2.4.2 Forage fish aggregation models

Using a hierarchical Bayesian framework, we developed a model to independently estimate abundance and size of FFA. The observed number of aggregations ($y_{\mathrm{ij}}$) per 4 km grid cell *i* and survey *j* generally followed a zero‐inflated negative binomial (ZINB) distribution due to overdispersion. We parameterized the ZINB using a zero‐inflated Poisson‐Gamma mixture formulation:

(1) $$y_{\mathrm{ij}}∼\text{Poisson}\left(\lambda_{\mathrm{ij}}\rho_{\mathrm{ij}}z_{\mathrm{ij}}\right)$$

(2) $$\begin{array}{c} \rho_{\mathrm{ij}}∼\text{Gamma}\left(r,r\right) \end{array}$$

(3) $$\begin{array}{c} z_{\mathrm{ij}}\sim \text{Bernoulli}\left(\psi_{\mathrm{ij}}\right), \end{array}$$

where $\rho_{\mathrm{ij}}$, is the dispersion parameter and $z_{\mathrm{ij}}$ is the zero‐inflation parameter, which models the realized FFA abundance, given the presence $\psi_{\mathrm{ij}}$, following a Bernoulli distribution.

We incorporated the season (*sea*1 = autumn, *sea*2 = spring, *sea*3 = winter) of survey *j* as a fixed covariate (categorical) on the parameter ($\psi_{\mathrm{ij}}$), using a logit‐linear link:

(4) $$\begin{array}{c} \text{logit}\left(\psi_{\mathrm{ij}}\right)=\alpha_{1}+\beta_{\mathrm{sea}1}x_{\mathrm{sea}1_{\mathrm{ij}}}+\beta_{\mathrm{sea}2}x_{\mathrm{sea}2_{\mathrm{ij}}}+\beta_{\mathrm{sea}3}x_{\mathrm{sea}3_{\mathrm{ij}}}. \end{array}$$

The log of expected mean abundance ($\lambda_{\mathrm{ij}}$) was modeled as the linear predictor, $\log\left(\theta_{\mathrm{ij}}\right)$, with survey effort ($e_{\mathrm{ij}}$) added as an offset:

(5) $$\begin{array}{c} \log\left(\lambda_{\mathrm{ij}}\right)=\log\left(\theta_{\mathrm{ij}}\right)+e_{\mathrm{ij}}.\; \end{array}$$

We modeled the linear predictor $\log\left(\theta_{\mathrm{ij}}\right)$ as a log‐linear equation with a spatial random effect (${u_{1}}_{\mathrm{ijk}}$) at the 32 km grid cell (*k*) scale to account for spatial autocorrelation, and a suite (*n* = 10) of environmental covariates (*l* = 1, 2, … *n*; Table 2):
(6)

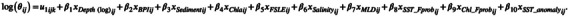




The spatial random effect (${u_{1}}_{\mathrm{ijk}}$) was incorporated as a proper Gaussian conditional autoregressive (CAR) model (Banerjee et al., 2003; Besag et al., 1991) that accounts for spatial dependence of grid‐level data. The CAR model follows a multivariate normal distribution parameterized in terms of covariance:

(7) $$\begin{array}{c} {u_{1}}_{\mathrm{ijk}}\sim \mathrm{MVN}\left(\mu_{\mathrm{CAR}},\frac{1}{\tau_{\mathrm{CAR}}}{\left(I-\gamma C\right)}^{-1}M\right), \end{array}$$

where ***I*** is the identity matrix, ***C*** is the normalized weight matrix, and ***M*** is the diagonal matrix of conditional variances, $\tau_{\mathrm{CAR}}$ is the precision scalar and *γ* is the degree of spatial dependence.

We modeled the logged observed aggregation size ($\log\left(s_{a}\right)$) for aggregation *a* as a Gaussian distribution:

(8) $$\begin{array}{c} \log\left(s_{a}\right)\sim \text{Gaussian}\left(\mu_{{\text{Size}}_{a}},\tau_{{\text{Size}}_{a}}\right), \end{array}$$

where $\mu_{{\text{Size}}_{a}}$ is the mean and $\tau_{{\text{Size}}_{a}}$ is the variance of $\log\left(s_{a}\right)$. The estimated aggregation size ($\mu_{{\text{Size}}_{a}}$) was modeled using a linear equation, incorporating a second spatial CAR set up as above (${u_{2}}_{\mathrm{ak}}$) as a random effect and the same suite of environmental covariates (*l* = 1, 2, … *n*):

(9) $$\begin{array}{c} \mu_{{\text{Size}}_{a}}={u_{2}}_{\mathrm{ak}}+\left(\sum_{l=1}^{n}{\beta_{\text{Size}}}_{l}x_{\mathrm{al}}\right). \end{array}$$

For consistency and to facilitate comparisons, the FFA abundance and size models were fit with the SST and Chl*a Fprob* frontal metrics to match the best‐performing community model (see Section 3). In the FFA models, we chose SST seasonal anomaly and benthic position index (BPI), instead of SST and rugosity, respectively, due to multicollinearity at the FFA study scale.

We used the R package NIMBLE (version 0.12.2, r‐nimble.org, de Valpine et al., 2017) to specify the FFA models and run the MCMC simulations. Three chains were sampled for 400,000 iterations each with 250,000 iterations discarded as burn‐in and thinned by 20, giving 7500 posterior samples per chain. As with the HMSC models, chain convergence was assessed visually and using the PSRFs. Priors were selected to be minimally informative to achieve convergence of the MCMC algorithm. For additional information on model specification, see https://github.com/cgoetsch/Forage_Fish_Aggregation_Models.

To evaluate model fit, we conducted a posterior predictive check of both the abundance and size models (Gelman et al., 2013; Kery & Royle, 2016), calculating the Chi‐square discrepancy measure (Freeman‐Tukey Goodness‐of‐Fit) and the Bayesian *p*‐values. As a measure of explanatory power, we calculated the correlations between the observed data and predictions from the model fit to all data. To assess internal model consistency and predictive accuracy, we conducted a fivefold cross‐validation with the data randomly assigned to folds. The correlation between observed data and model predictions (in‐sample and out‐of‐sample) was calculated for each fold and summarized (mean ± standard deviation) across the five folds. Pearson correlation was used for the abundance model and Spearman correlation was used for the size model to account for a few extreme outliers in the predictions.

### 2.5 Model predictions

#### 2.5.1 Forage fish community models

To identify the important environmental drivers of forage fish community occupancy for each season (autumn and spring), we calculated the mean estimates of the beta parameters (fixed effects) with significant drivers defined as those with at least 95% posterior probability. We summarized the beta parameters, calculating the mean absolute parameter values (hereafter, effect size) to evaluate the overall covariate relationship strength. We also predicted seasonal species‐specific occurrence probability over the 4 km grid expanded to encompass the NES study area, using the seasonal means of the environmental covariates. From the predicted occurrence probabilities, we identified distinct community types within each season using *k*‐means cluster analysis to classify species composition patterns over space (kmeans in stats package; Foster et al., 2013; Ovaskainen & Abrego, 2020). We determined the optimal number of clusters using the elbow method (NbClust package, Charrad et al., 2014) and calculated the prevalence (i.e., mean occurrence probability) of each species within the community types. We also calculated the seasonal species richness (i.e., summed multispecies probability of occurrence per grid cell) across the NES study area and the FFA study area and calculated the species richness mean ± standard deviation for each community type by season for both study areas.

#### 2.5.2 Forage fish aggregation models

To determine which covariates were important drivers of FFA abundance and size, we calculated the median and 95% credible intervals (CI) of the fixed effects for each model. We evaluated how the most important environmental drivers influenced FFA abundance and size by calculating predictions across an environmental gradient (i.e., the maximum and minimum values of those covariates from the observed data). We also made seasonal (autumn, winter, spring, summer) predictions of abundance, size, and surface availability (abundance × size) over the 4 km FFA grid, using the seasonal means of the environmental covariates over our study period. In addition, we calculated the FFA abundance (mean ± standard deviation) and FFA density (FFA/km^2^) per community type (as defined from the community models) within the FFA study area for autumn and spring.

## 3 RESULTS

During the aerial digital survey study period (2012–2019), there were a total of 21,934 surface FFAs (New York project: 14,288, Mid‐Atlantic project: 7646) observed in the combined FFA study area (Figure 1a). Of these, most FFAs were observed in the summer (16,667) and the autumn (5085) compared to the spring (175) and winter (7). The size of aggregations ranged from 0.5 to 8651.2 m^2^ with a mean size of 96.5 m^2^.

### 3.1 Model performance

#### 3.1.1 Forage fish community models

For both seasons, the *Fprob* model was a marginally better fit than the *Fmean* model as evaluated by the WAIC scores (Table 3); thus, further discussion of the *Fmean* models is not presented. The autumn and spring *Fprob* models showed a good fit to the data with mean AUC scores of explanatory power of >0.93 for both (Table 3). Overall, the autumn *Fprob* model had high predictive accuracy with a 0.882 mean cross‐validated AUC, while the spring model had lower predictive accuracy with a mean cross‐validated AUC of 0.795. The species‐specific predictive accuracy for autumn ranged from 0.761 for butterfish to 0.955 for thread herring (Table 3). The predictive accuracy of individual species in the spring had greater variation; AUC scores ranged from 0.577 (mackerel) to 0.940 (silverside; Table 3). As with the autumn models, all species in the spring had acceptable fit. Including species associations marginally increased conditional predictive accuracy for autumn (mean AUC: 0.892, species‐specific AUC range: 0.764–0.971; Table 3). For the spring models, including species associations greatly improved predictive accuracy (mean conditional AUC: 0.844 and species‐specific AUC: 0.723–0.939) with the greatest increases seen for species with low initial predictive accuracy.

**TABLE 3:** Fit statistics and cross‐validation results for the forage fish community models.

Model	Species	WAIC	Explanatory power	Predictive accuracy	Conditional predictive accuracy
Autumn *Fprob*	‐	2.305	0.949	0.882	0.892
	alewif		0.986	0.927	0.945
	atherr		0.989	0.955	0.971
	atlher		0.971	0.942	0.946
	atlmac		0.949	0.845	0.866
	atlmen		0.964	0.920	0.927
	atsaur		0.891	0.795	0.796
	bayanc		0.985	0.945	0.954
	bluher		0.989	0.923	0.954
	butter		0.886	0.761	0.777
	rherri		0.893	0.814	0.804
	sandla		0.855	0.766	0.764
	silanc		0.953	0.854	0.863
	spsard		0.981	0.946	0.959
	stranc		0.992	0.954	0.966
Autumn *Fmean*	‐	2.307	‐	‐	‐
Spring *Fprob*	‐	2.659	0.937	0.795	0.844
	alewif		0.944	0.723	0.802
	atlher		0.929	0.634	0.776
	atlmac		1.000	0.577	0.723
	atlmen		0.921	0.900	0.902
	atlsil		0.949	0.940	0.939
	bayanc		0.947	0.934	0.935
	bluher		0.986	0.701	0.805
	butter		0.873	0.825	0.830
	sandla		0.853	0.801	0.808
	stranc		0.971	0.917	0.917
Spring *Fmean*	‐	2.663	‐	‐	‐

*Note*: The mean and species‐specific AUC scores for explanatory power (model fitted with all data), predictive accuracy (threefold cross‐validation), and conditional predictive accuracy (conditional cross‐validation) are provided for the best‐performing model of each season.

Abbreviations: AUC, area under the receiver operator curve; *Fmean*, indicates model including the Chl and SST *Fmean* covariates; *Fprob*, indicates model including the Chl and SST *Fprob* covariates; WAIC, Watanabe‐Akaike information criterion.

#### 3.1.2 Forage fish aggregation models

**FIGURE A2:**
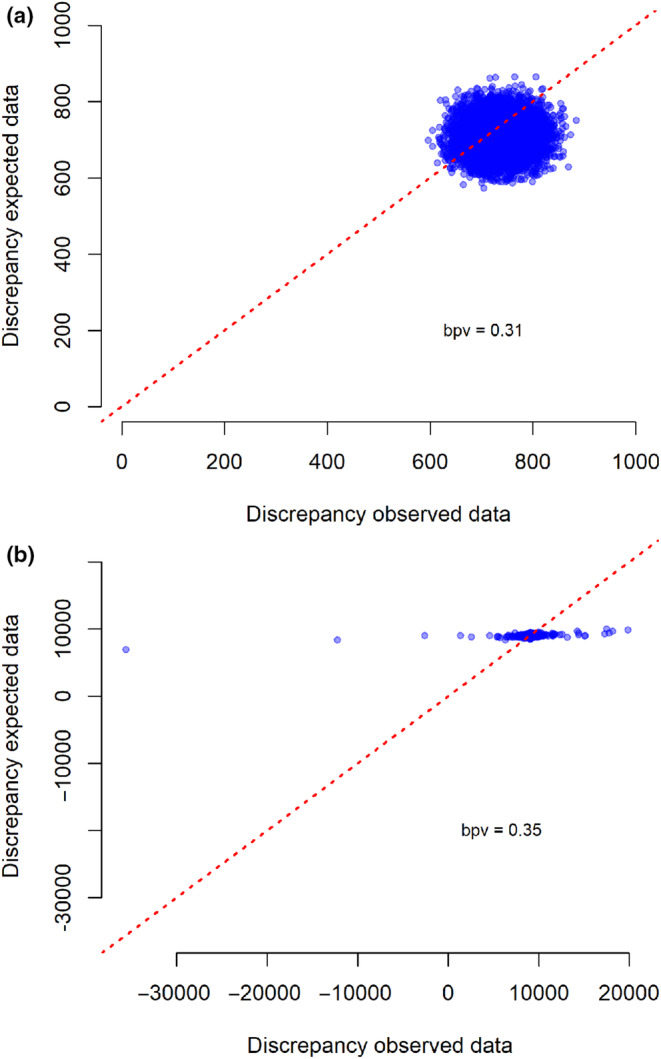
Posterior predictive check for (a) abundance and (b) size forage fish aggregation models, showing the observed versus expected Chi‐square discrepancy measures (Freeman–Tukey goodness‐of‐fit) and the calculated Bayesian *p*‐values (bpv).

The posterior predictive check showed appropriate model specification for both the abundance and size models with Bayesian *p*‐values of .31 and .35, respectively (Table 4; Appendix 2: Figure A2). The explanatory power, as calculated from the correlations between the observed and predicted values for the full model, indicated an adequate fit for the abundance model (0.23 correlation) and a good fit for size model (0.59). Correlations were also consistent across the in‐sample cross‐validation for both models (abundance: 0.23 ± 0.002; size: 0.59 ± 0.008; Table 4). The out‐of‐sample predictive accuracy (Table 4) was similar to the explanatory power for both abundance (0.22 ± 0.007) and size (0.49 ± 0.08) models, also showing consistency across folds. The primary aim of the FFA model was to conduct inference with prediction as a secondary priority; model performance meets that goal. The lower out‐of‐sample predictive accuracy suggests that extrapolations from this model would have less value than within‐sample prediction.

**TABLE 4:** Fit statistics and cross validation results for the forage fish aggregation (FFA) models.

Model	Bvp	Explanatory power	In‐sample predictive accuracy (mean ± SD)	Out‐of‐sample predictive accuracy (mean ± SD)
Abundance	0.31	0.23	0.23 ± 0.002	0.22 ± 0.007
Size	0.35	0.59	0.59 ± 0.008	0.49 ± 0.08

*Note*: Abundance and size models were evaluated using a posterior predictive check with a Freeman–Tukey Goodness‐of‐Fit test, giving the Bayesian *p*‐value (bpv). The correlation between observed data and model estimates was calculated as a measure of explanatory power (full model) and predictive power (fivefold cross‐validation: in‐sample and out‐of‐sample). Pearson correlation was used for the abundance model, while Spearman was used for the size model due to a few extreme outliers in model estimates.

### 3.2 Environmental drivers

For the community models, the environmental covariates accounted for most of the explained variance (autumn mean: 75.0%; spring mean: 62.6%), followed by the tow‐level random effect (autumn mean: 15.4%; spring mean: 27.8%) and temporal random effect (mean: 9.6% for both). Within the fixed covariates, the dynamic surface covariates were the most explanatory in the autumn (41.6%), followed by the static covariates (31.0%), while in the spring the static covariates (31.2%) and dynamic surface covariates (29.7%) were similarly explanatory.

**FIGURE 2:**
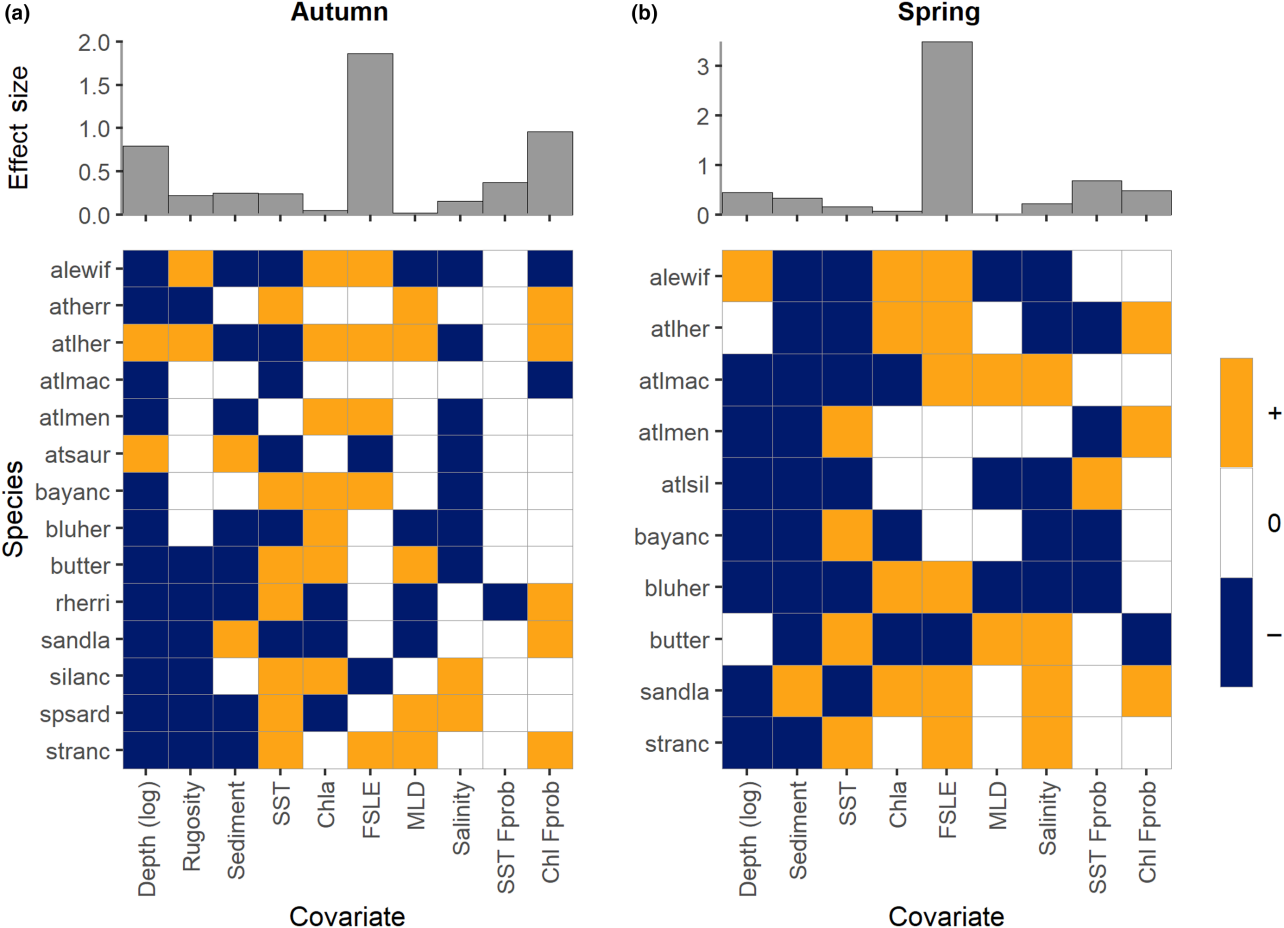
Beta parameter estimates for the (a) autumn and (b) spring community models. Orange and blue grid squares represent a significant relationship (either positive or negative, respectively) between the probability of occupancy and each environmental covariate. White grid cells represent non‐significant relationships. Beta parameters were considered significant if they had at least 95% posterior support. The top panels depict the community‐level covariate effect size, calculated as the mean absolute parameter values.

Important drivers for most of the community across both seasons were the static habitat feature depth (autumn: all spp.; spring: 8 of 10 spp) and the dynamic surface feature SST (autumn: 13 of 14 spp; spring; all spp; Figure 2). In autumn, depth had the third highest effect size (0.80; Appendix 2: Table A2a), while SST only had a moderate effect size (0.24). All species, except herring and saury, had a negative relationship with depth, so as depth increased the probability of occurrence decreased (Figure 2a). In autumn, the probability of occurrence increased as SST became warmer for seven species and decreased with warmer SST for six species; only menhaden had a non‐significant relationship with SST (Figure 2a). In spring, depth had a lower effect size (0.44), and all significant species except alewife had decreased probability of occurrence with increasing depth. The probability of occurrence increased with warmer SST for only four species in the spring (Figure 2b), while occurrence probability decreased with warmer SST for the remainder. For both seasons, MLD (i.e., mixed layer depth, an indicator of seasonal stratification) had the lowest effect size (0.02 and 0.01 for autumn and spring, respectively; Appendix 2: Table A2) but was still a significant driver for part of the community (autumn: 9 of 14 spp; spring: 5 of 10 spp; Figure 2).

In autumn, the dynamic surface features, FSLE (i.e., presence of submesoscale eddies and filaments) and Chl *Fprob* (i.e., productivity front persistence) had the highest effect size (1.87 and 0.96, respectively, Appendix 2: Table A2a) but were only significant drivers of occurrence for about half the community (7 spp each; Figure 2a). In the spring, the covariates with the highest effect size were FSLE (3.49) and SST *Fprob* (0.69; Figure 2b and Appendix 2: Table A2b). While FSLE was significant for seven species during spring, SST *Fprob* was only a significant driver for five species (Figure 2b). For most species, FSLE had a positive relationship with the probability of occurrence; only two species in the autumn (saury and silver anchovy) and one in the spring (butterfish) were negatively associated with FSLE. Chl *Fprob* had a mostly positive relationship with occurrence (5 of 7 spp); only alewife and mackerel showed the opposite relationship (Figure 2a). In the spring, SST *Fprob* had a mostly negative relationship with occurrence (4 of 5 spp; Figure 2b).

**FIGURE 3:**
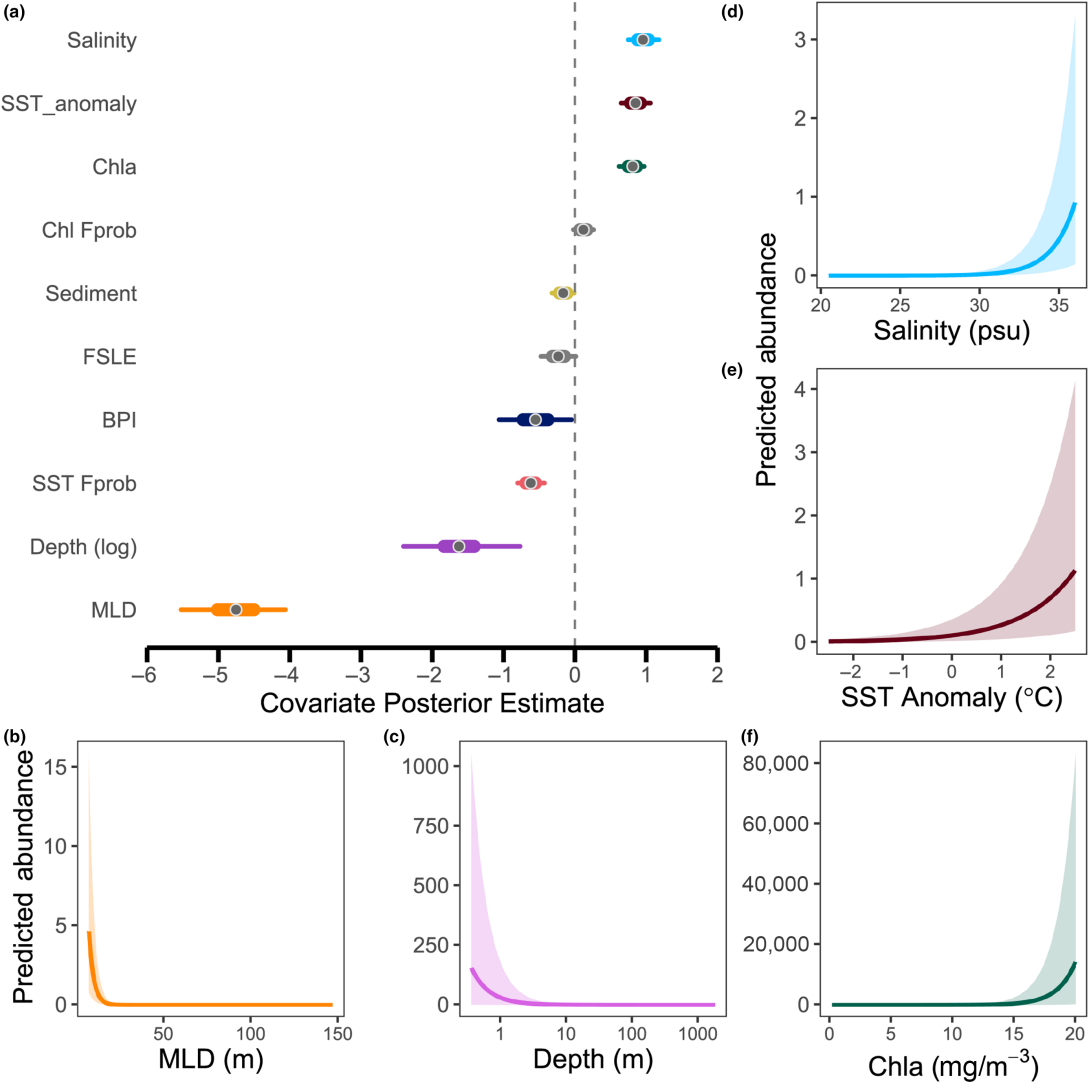
(a) Beta parameter estimates and credible intervals (CIs) from the forage fish aggregation (FFA) abundance model. Gray points represent the parameter medians, thick lines the 50% CI, and thin lines the 95% CI. Parameters with gray CIs are not significant. (b–f) FFA abundance predictions relative to the five strongest beta parameters. Shaded areas represent the 95% CI of the estimate.

**FIGURE 4:**
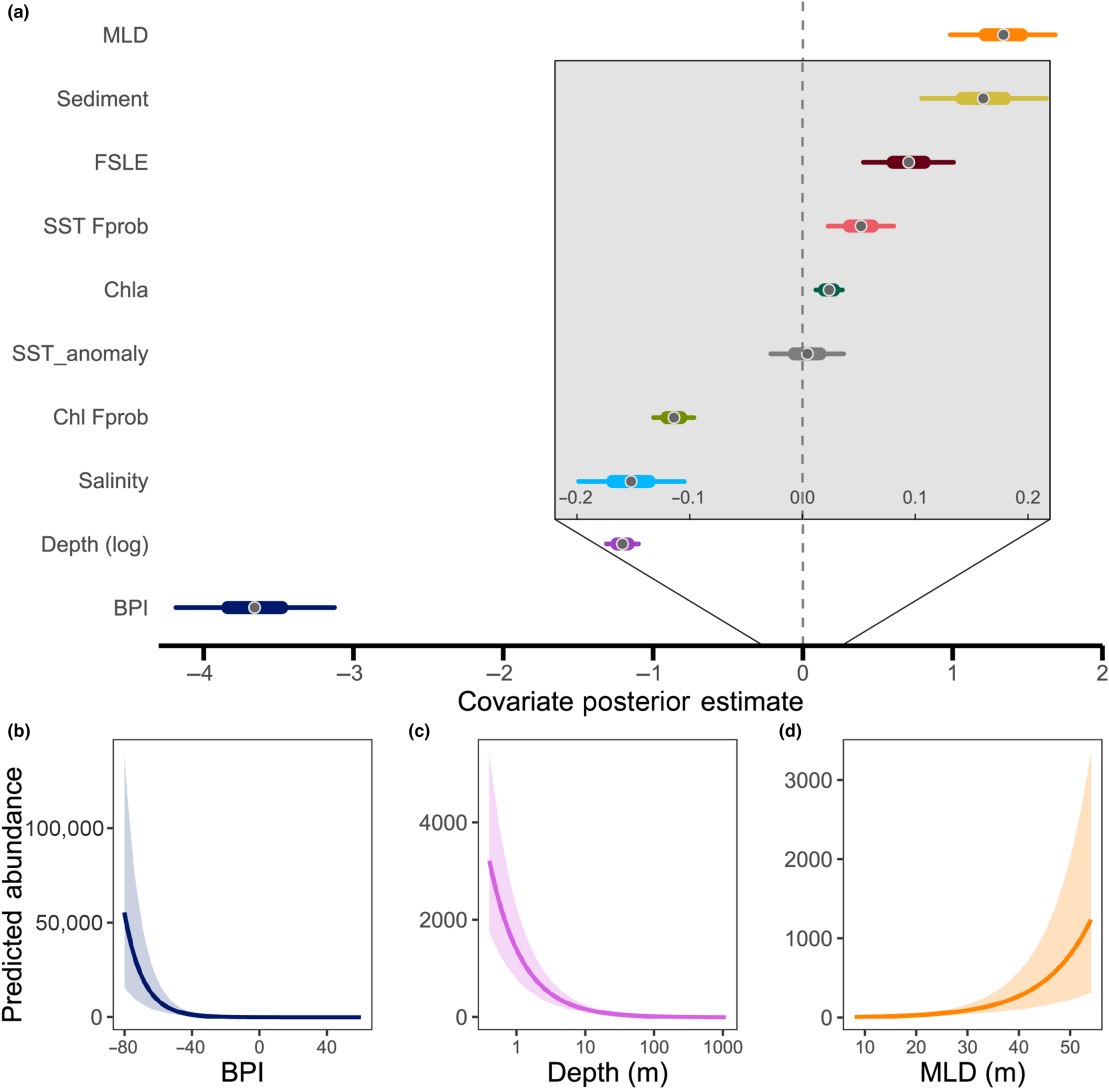
(a) Beta parameter estimates and credible intervals (CI) from the forage fish aggregation (FFA) size model. Points represent the parameter medians, thick lines the 50% CI, and thin lines the 95% CI. The inset (gray box) enlarges the scale for parameters close to zero to improve readability. Parameters with gray CIs are not significant. (b–d) FFA size predictions relative to the three strongest beta parameters. Shaded areas represent the 95% CI of the estimate.

In both the FFA abundance and size models, the majority of environmental covariates had statistically significant effects (i.e., 95% CIs did not contain 0; Figures 3 and 4). The most important covariates for estimating abundance were MLD, depth, salinity, SST anomaly, and Chl*a* (Figure 3). As MLD and depth decreased, FFA abundance increased. Conversely, as salinity, SST anomaly, and Chl*a* increased, so did FFA abundance. Although Chl*a* had the lowest absolute parameter estimate of these five covariates, the predicted median abundance across the range of Chl*a* in the study area was two orders of magnitude higher than median abundance across the range of depth, and four orders of magnitude higher than for MLD, salinity, and SST anomaly. Only Chl *Fprob* and FSLE were not significant for abundance.

The most important covariates for estimating FFA size were BPI, depth, and MLD (Figure 4). BPI had a strong negative effect on estimated FFA size but was most influential in the −80 to −40 range which corresponds to steep crevasses or valleys. Higher values of BPI corresponding to bathymetric flats (near 0) and steep hills or peaks (>1), were associated with very low FFA size. As depth increased, the estimated size of aggregations decreased. MLD had opposite effects on abundance versus size: as MLD increased (i.e., the water column became less stratified), there were fewer, but larger, FFAs. SST anomaly was the only non‐significant covariate for aggregation size.

### 3.3 Spatial and seasonal patterns

**FIGURE 5:**
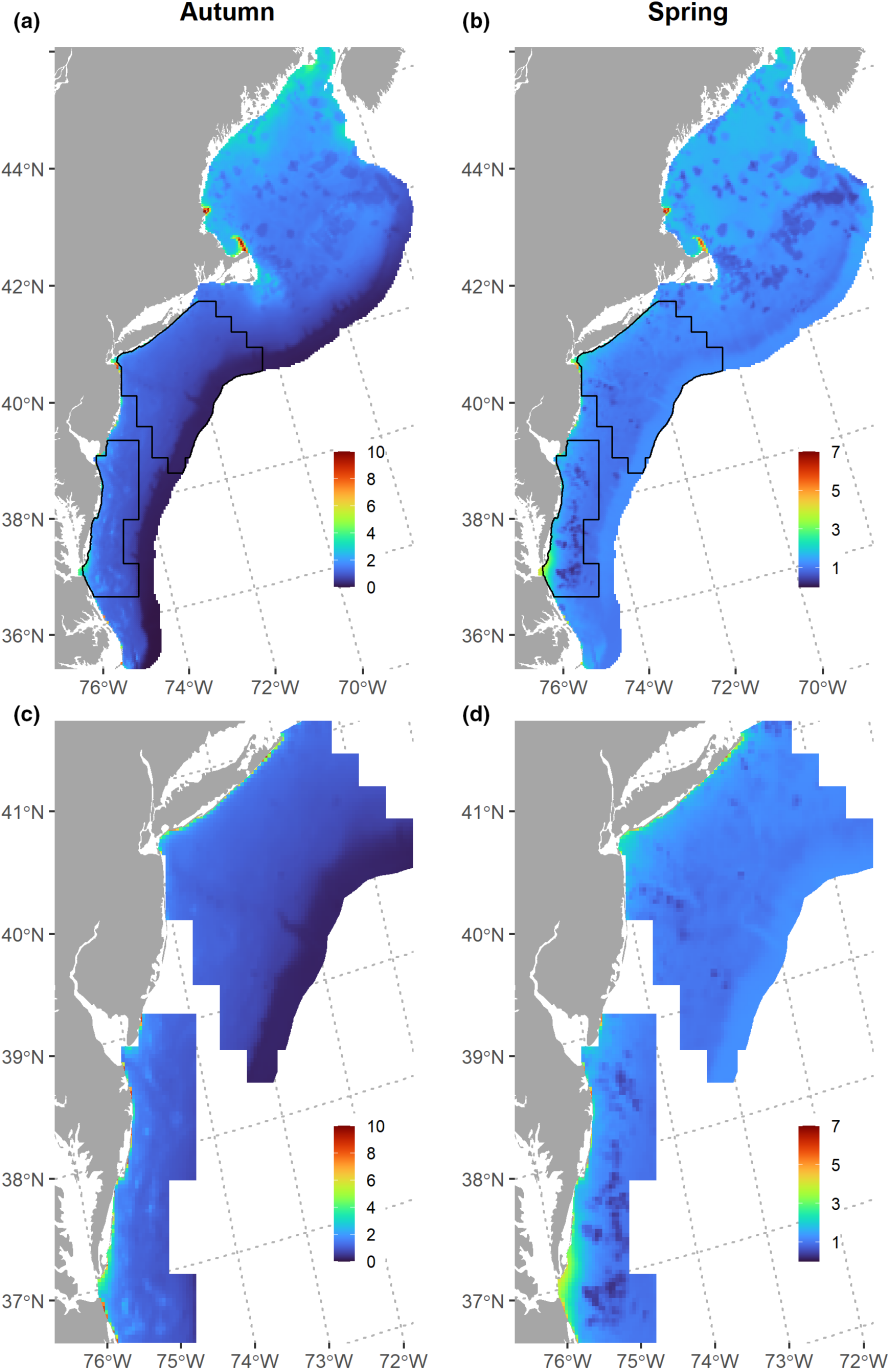
Forage fish community species richness for (a, c) autumn and (b, d) spring across the Northeast Continental Shelf (NES) study area. Black outlines (a, b) delineate the forage fish aggregation (FFA) study area. The bottom panels enlarge the FFA study area. Species richness was calculated as the summed probability of occurrence across all species. Note that the scales for autumn and spring differ due to differences in the maximum species richness possible for each season.

**FIGURE 6:**
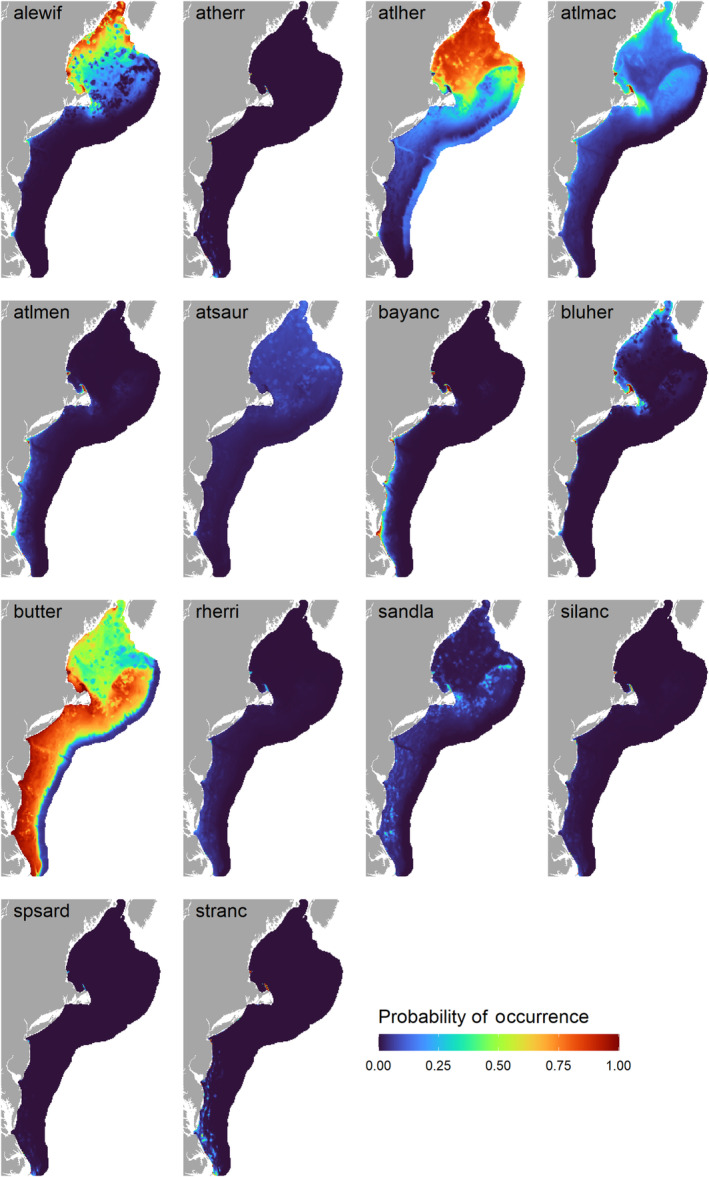
Autumn community model predictions of forage fish occurrence, based on bottom trawl data. Species codes are defined in Table 1.

**FIGURE 7:**
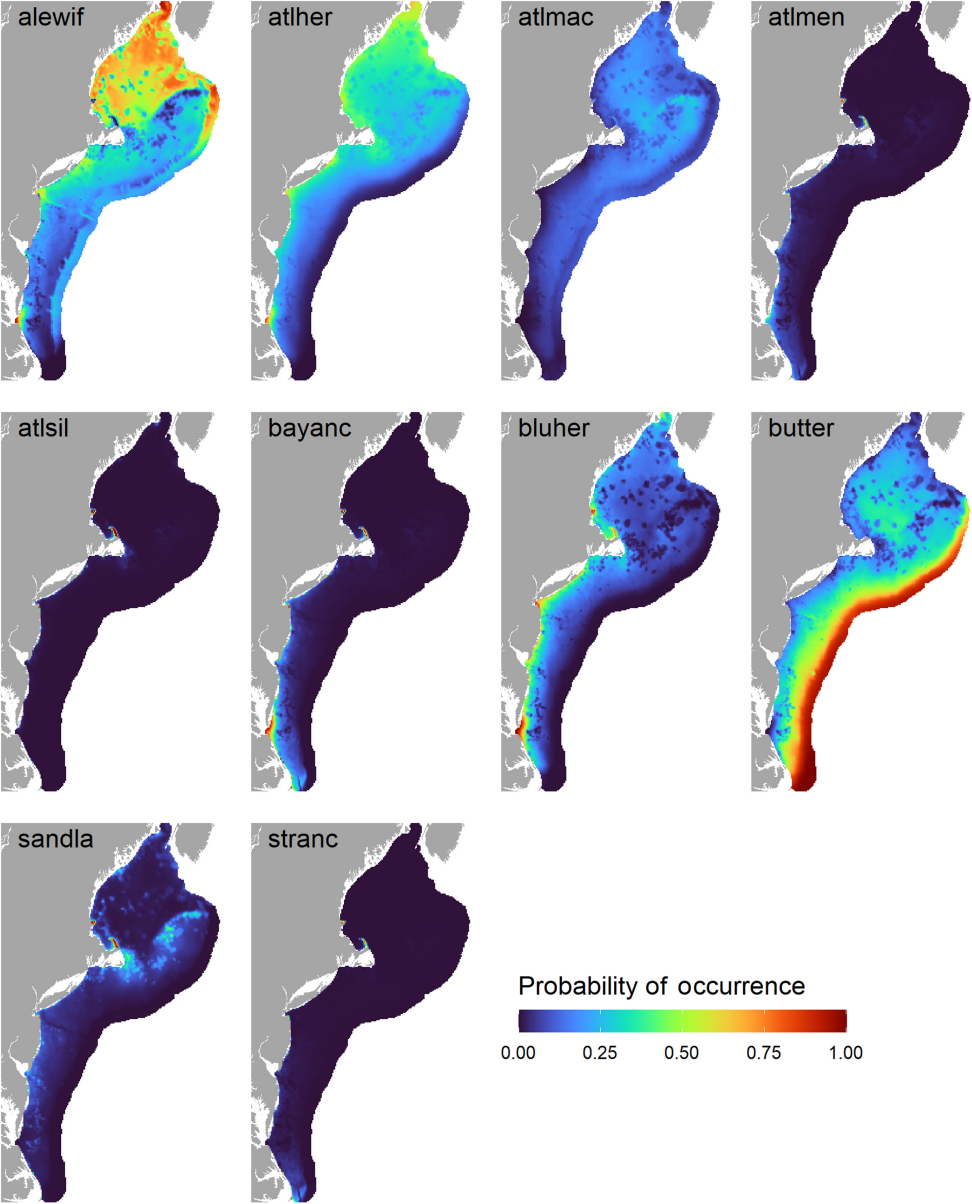
Spring community model predictions of forage fish occurrence, based on bottom trawl data. Species codes are defined in Table 1.

The predicted species richness (i.e., summed occupancy) from the community models was highest nearshore and in the Gulf of Maine in the autumn; in spring, species richness was lower overall and less variable across the study area (Figure 5). In general, the predicted occurrence distributions of forage fishes in autumn, except for butterfish, were more concentrated either nearshore or in the Gulf of Maine (Figure 6). Conversely, the spring‐predicted occurrence distributions were more diffused across the shelf (Figure 7).

**FIGURE 8:**
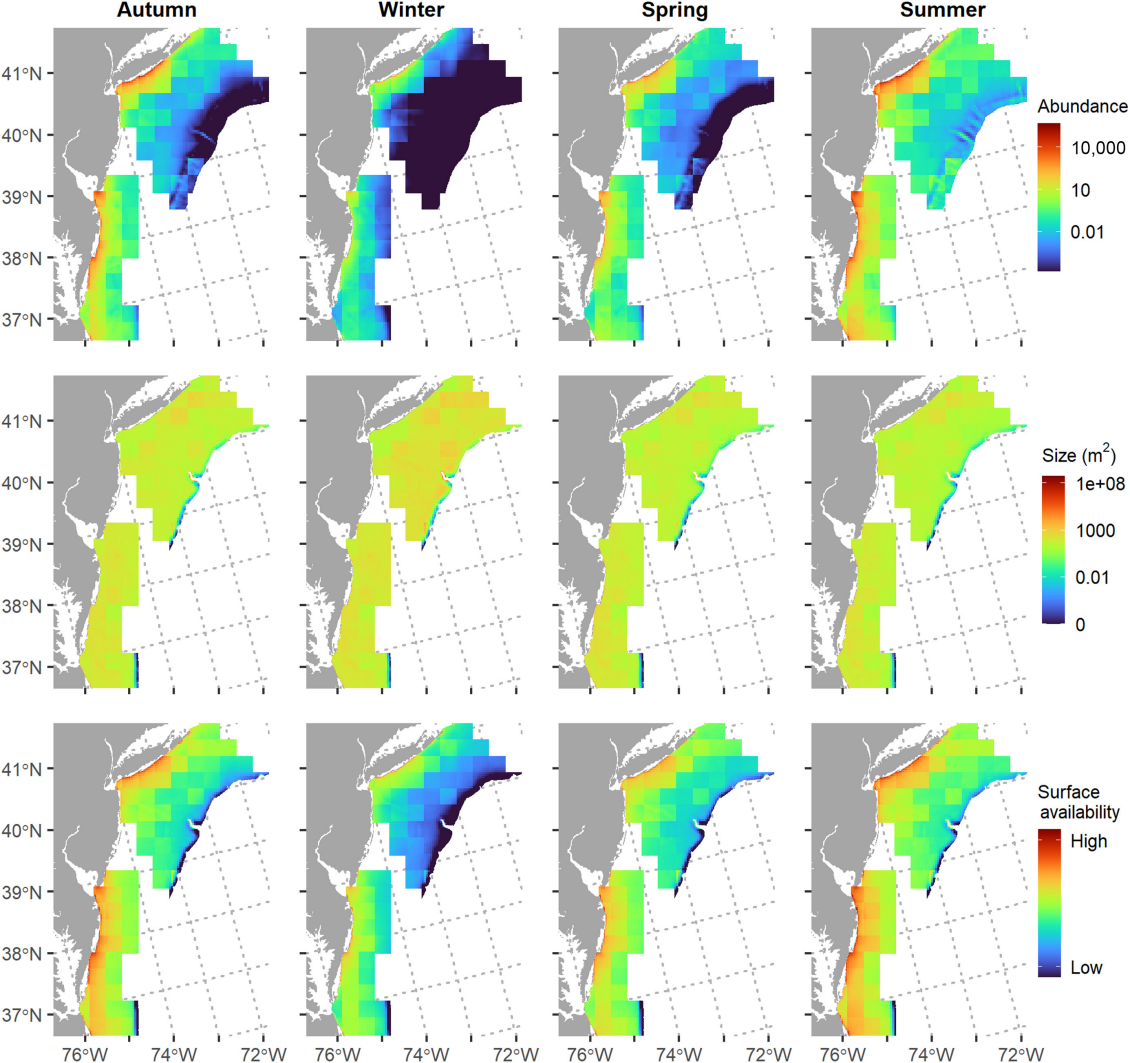
Predicted spatial distribution of forage fish aggregation (FFA) abundance, size, and surface availability (abundance × size, cumulative m^2^). The spatial extent of the size and availability predictions has been reduced to only include on‐shelf areas.

The FFA abundance model predicted the highest number of aggregations in the nearshore areas off the coasts of Delaware, Maryland, and Virginia, known as the Delmarva Peninsula and the southwestern end of Long Island (Figure 8). The areas of high FFA abundance were similar across all seasons, while the magnitude of abundance varied from highest in the summer to lowest in the winter. Across the mid‐to‐outer shelf in the FFA study area, there were consistently low counts (1–10) of FFAs even during the summer, and almost no FFAs predicted in those areas during the winter. Autumn had a higher abundance of predicted FFAs than spring, particularly nearshore.

In general, the predicted size of aggregations was more spatially uniform than abundance, with smaller FFAs predicted near the continental shelf break. The predicted size of aggregations was larger in the winter than in the other seasons, corresponding to less shelf stratification (i.e., deeper MLD). Since the predicted size was fairly constant across much of the shelf, the surface availability (abundance × size) followed similar spatial patterns to that of FFA abundance.

### 3.4 Forage fish community types

**FIGURE 9:**
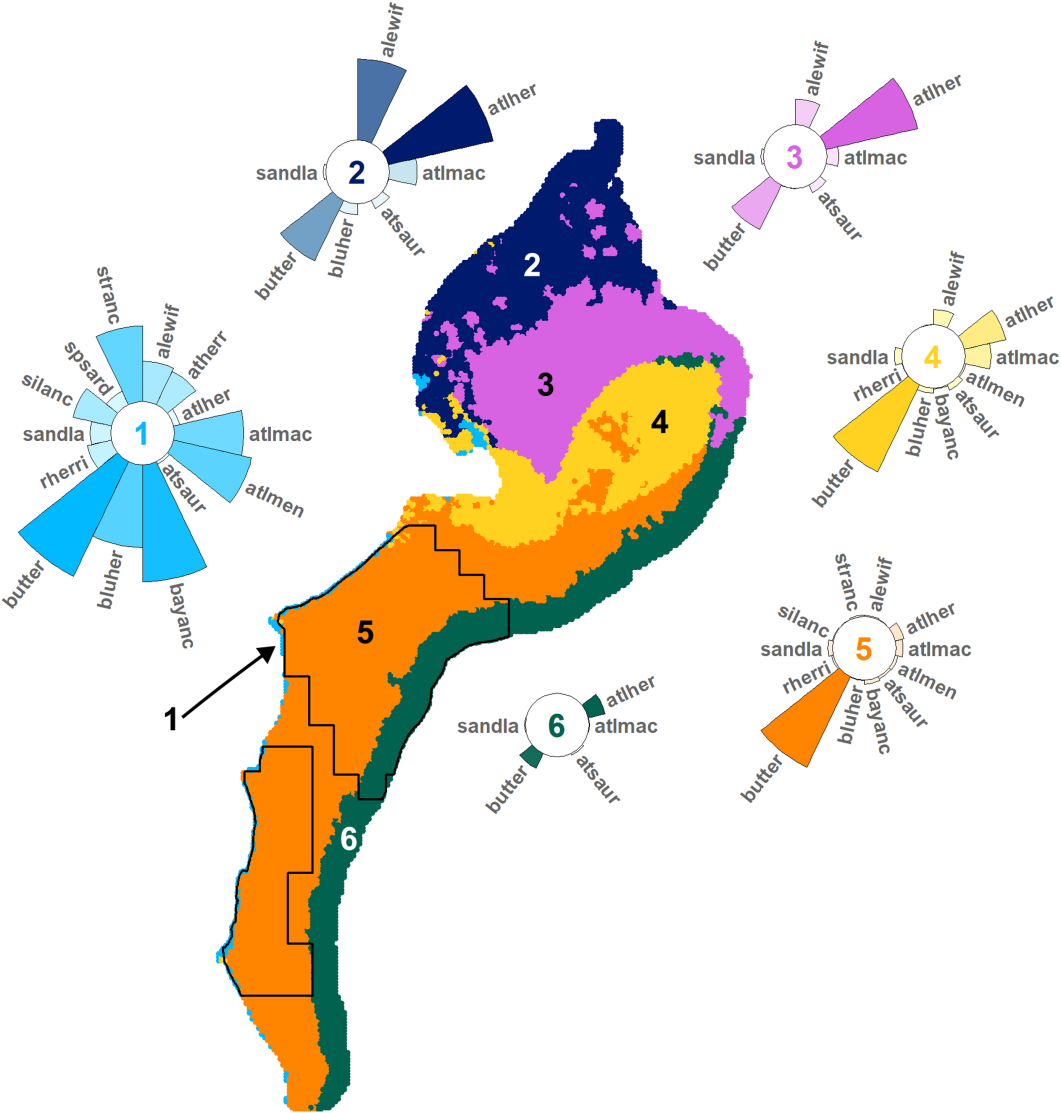
Autumn forage fish community types: six distinct forage fish community types were identified across the NES study area via *k*‐means cluster analysis. Circular bar plots depict the prevalence (i.e., mean occurrence probability) of species within the community type (*y*‐scale max = 1). Species codes are defined in Table 1. Species with a prevalence <0.003 are not shown. Black outlines define the forage fish aggregation (FFA) study area. See Appendix 2: Table A3a for prevalence estimations. NES – U.S. Northeast Continental Shelf.

**FIGURE 10:**
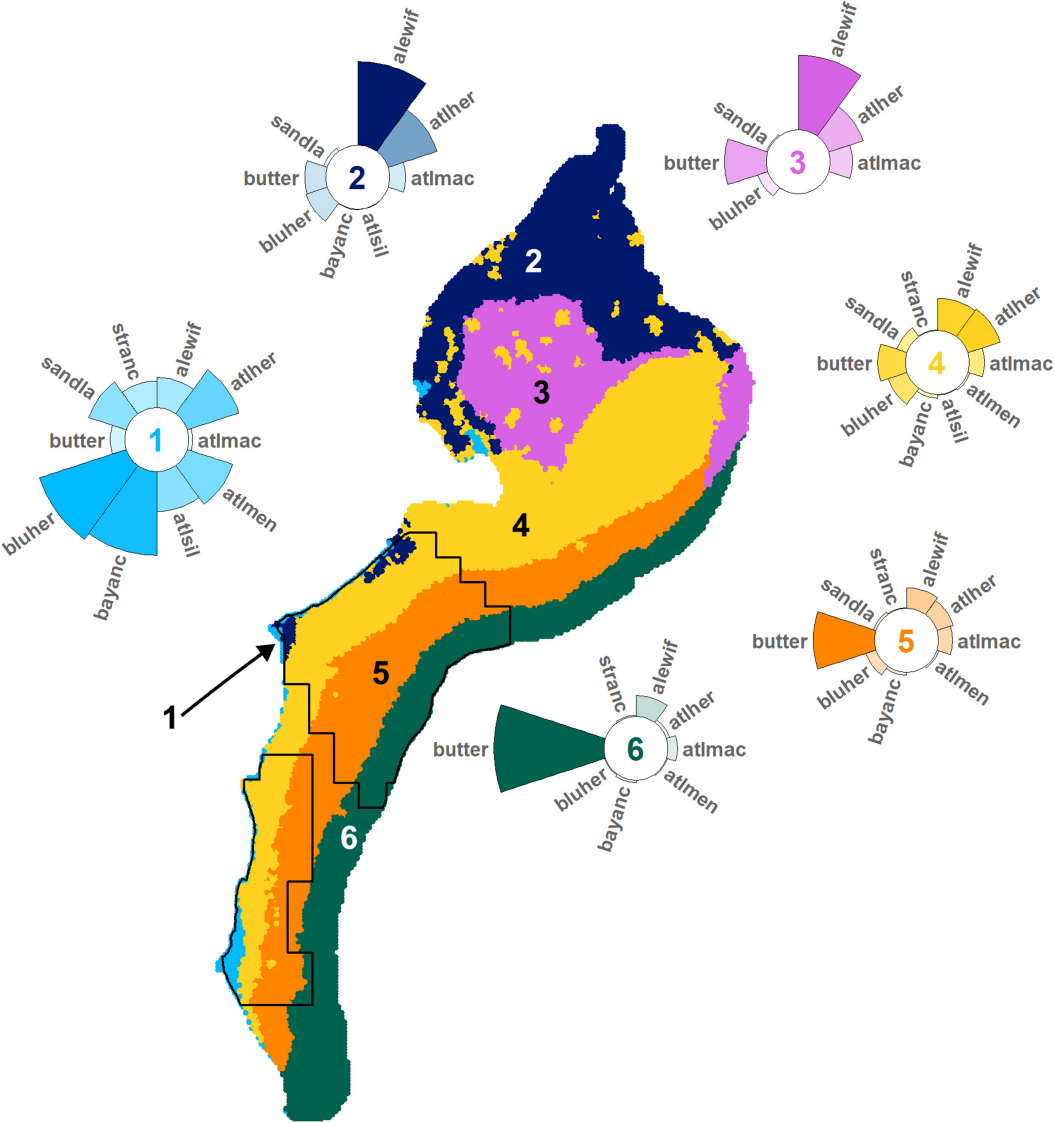
Spring forage fish community types: six distinct community types were identified across the NES study area via *k*‐means cluster analysis. Circular bar plots depict the prevalence (i.e., mean occurrence probability) of species within the community type (*y*‐scale max = 1). Species codes are defined in Table 1. Species with a prevalence <0.003 are not shown. Black outlines define the forage fish aggregation study area. See Appendix 2: Table A3b for prevalence estimations. NES – U.S. Northeast Continental Shelf.

From the *k*‐means cluster analysis of the community occupancy predictions, we found six distinct forage fish community types in the NES study area for both seasons (Figures 9 and 10). The community types roughly correspond with broadscale features across the NES. For both seasons, Community Type 1 is located nearshore, particularly in the New York and Mid‐Atlantic Bights but also in a few areas in the coastal Gulf of Maine. Community Types 2 and 3 represent the Gulf of Maine Coastal Current and the Gulf of Maine Basins areas, respectively, although the spatial extent varies between seasons. Moreover, in the spring, Community Type 2 also appears in the New York Bight. Community Type 4 covers the Nantucket Shoals and Georges Banks areas in both seasons; however, in spring, this community type also includes a strip of the inner continental shelf across the New York and Mid‐Atlantic Bights. In the autumn, Community Type 5 encompasses most of the continental shelf across the New York and Mid‐Atlantic Bights; while, in the spring, it only covers the outer shelf. Community Type 6 covers the area of the continental slope in both seasons.

For both seasons, Community Type 1 had the highest mean species richness (autumn: 5.79; spring: 3.37; Table 5) with 12 of 14 species in the autumn and 9 of 10 species in the spring having prevalence >10% (Figures 9 and 10, Appendix 2: Table A3). Community Type 2 had the second highest species richness (autumn: 2.41; spring: 1.56; Table 5). Herring dominated Communities 2 and 3 in the autumn, while in the spring, alewife was more prevalent. Community Types 4 and 5 were butterfish‐dominated in the autumn (Figure 9); whereas in the spring, Community Type 4 was not dominated by any species and Community Type 5 remained butterfish‐dominated (Figure 10). During the autumn, Community Type 6 had the lowest species richness (0.28) with most forage fish species uncommon; while in the spring, Community Type 5 had the lowest species richness (1.03) and was butterfish‐dominated.

**TABLE 5:** Species richness, forage fish aggregation (FFA) abundance, and FFA density by community type across the US Northeast Continental Shelf (NES) and FFA study areas.

Season	Community type	NES richness	FFA area richness	FFA abundance	FFA density
Autumn	1	5.79 ± 1.89	5.63 ± 1.86	14,441.17 ± 43,985.09	1917.49
	2	2.41 ± 0.38	‐	‐	‐
	3	1.50 ± 0.28	‐	‐	‐
	4	1.58 ± 0.39	1.92 ± 0.60	132.11 ± 447.95	9.11
	5	1.07 ± 0.34	1.11 ± 0.35	27.52 ± 231.63	1.72
	6	0.29 ± 0.18	0.29 ± 0.29	0 ± 0.00	0.0
Spring	1	3.37 ± 1.07	3.22 ± 0.97	634.72 ± 2174.49	59.09
	2	1.56 ± 0.21	1.65 ± 0.24	9.41 ± 19.40	0.61
	3	1.46 ± 0.14	‐	‐	‐
	4	1.15 ± 0.25	1.24 ± 0.27	11.20 ± 55.09	0.70
	5	1.03 ± 0.13	1.01 ± 0.13	0.13 ± 0.36	0.01
	6	1.17 ± 0.14	1.17 ± 0.10	0 ± 0.00	0.00

*Note*: Species richness was calculated for each community type as the mean ± SD of the summed occupancy for all grid cells classified to that type. FFA abundance metrics were calculated for the community types represented in the FFA study area. FFA abundance is the estimated number (mean ± SD) of FFA spatially overlapping each community type. FFA density is calculated as the number of FFA per km^2^ for each community type.

Within the FFA study area, four community types were represented in the autumn, while during the spring, five were represented (Figures 9 and 10, Table 5). In both seasons, Community Type 1 had the highest FFA abundance (autumn: 14,441.17; spring: 634.72) and density (autumn: 1917.49/km^2^; spring: 59.09/km^2^), Community Type 4 had second highest FFA abundance (autumn: 132.11; spring: 11.20) and density (autumn: 9.11/km^2^; spring: 0.70/km^2^), and Community Type 6 had the lowest for both (Table 5).

### 3.5 Species co‐occurrence

**FIGURE A3:**
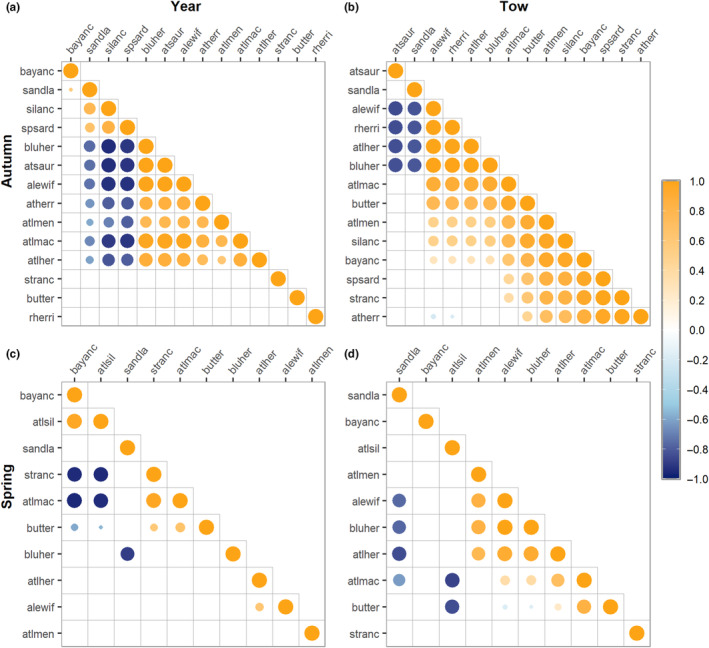
Residual species‐to‐species associations (i.e., species which co‐occur more or less than expected based on species' niches) in the (a, b) autumn and (c, d) spring community models. Panels (a) and (c) are due to the temporal random effect, while panels (b) and (d) are due to the tow random effect. Orange and blue indicate species pairs with at least 0.95 posterior support for a positive or negative association, respectively. The intensity of color and size of the marker indicates the strength of the association (in units of correlation). Species codes are defined in Table 1.

Species co‐occurrence patterns, representing the residual species‐to‐species associations from the random effects, showed correlations among species both temporally and by tow (Appendix 2: Figure A3). In the autumn, the temporal associations indicated that two groups of forage fishes fluctuate with each other over annual scales (Appendix 2: Figure A3a): (1) blueback herring, saury, alewife, thread herring, menhaden, mackerel, and herring; and (2) silver anchovy and Spanish sardine. Tow‐level associations in the fall showed complex co‐occurrence patterns (Appendix 2: Figure A3b). In the spring, there were few significant temporal or tow associations (Appendix 2: Figure A3c,d).

## 4 DISCUSSION

The community and aggregation patterns we found for NES forage fishes help fill an existing knowledge gap on oceanographic drivers and distribution of surface prey availability for upper trophic‐level marine predators in this ecosystem. Our community models identified potential hotspots of prey availability across the NES via species richness estimates, which overlapped spatially with areas of high FFA abundance in the New York and Mid‐Atlantic Bights. Examination of the patterns across the community and FFA models led to three cross‐cutting conclusions: (1) forage fish community models and FFA distributions indicate spatial overlap of hotspots, (2) static habitat features were important to both community and FFA distribution patterns, and (3) dynamic surface features were important drivers of community occupancy, while subsurface features were more important to FFAs.

### 4.1 Spatiotemporal patterns in forage fish availability

The predicted distribution of FFAs likely represents a more appropriate estimation of prey availability for surface‐feeding predators, since modeling surface aggregations incorporates a measure of patchiness driven by schooling behavior and vertical accessibility. Although spatially limited to the Mid‐Atlantic and New York Bight regions, our FFA models indicate increased abundance and total availability of surface FFAs in the nearshore areas off the southern coast of Long Island and the Delmarva Peninsula, with FFA abundance declining across the shelf to the continental slope. This result is consistent with abundance distributions of forage fishes' primary zooplankton prey, such as *Pseudocalanus* spp, *Temora longicornus*, and *Centropages typicus* (Kane & Prezioso, 2008; Suca, Deroba, et al., 2021), distributions of predators (Bangley et al., 2020; Goyert et al., 2018; Roberts et al., 2016), and commercial fishing effort for some of these forage fishes, which often target large surface schools (VMS Commercial Fishing Density Data, www.northeastoceandata.org; SEDAR, 2015).

Moreover, we found that areas of high predicted FFA abundance coincide with areas of high predicted species richness from the forage fish community models. In contrast, areas of high FFA abundance, or surface prey availability, do not always correlate with areas of high individual species occupancy. Based on this relationship, we would also expect high abundance of FFA in areas of high species richness in the NES outside the FFA study area (i.e., the Gulf of Maine, Nantucket Shoals, and Georges Banks). While we currently lack empirical data on FFA in these areas, they are known for high productivity and as important foraging areas for marine mammals and seabirds (Overholtz et al., 2007), suggesting that further study of the spatiotemporal patterns of FFA in these areas would inform our understanding of predator distributions and behaviors.

### 4.2 Joint species distribution models reveal forage fish community dynamics

Forage fish aggregation composition from aerial digital survey data cannot be identified to the species level, but the community models provide insight into which forage fish species likely compose FFAs across space. In the New York Bight in the spring, the area with the highest estimated abundance of FFAs overlapped with Community Type 1 of the community model. We can infer that there is a high likelihood of spring FFAs in this area being composed of blueback herring, bay anchovy, herring, or menhaden, with lower probabilities for other species in Community Type 1. In contrast, during the autumn in the same area, our models indicate there is a high likelihood that FFAs would be composed of butterfish or bay anchovy. More research is needed on the species composition of FFAs in these areas over time to validate these model predictions.

The residual species associations from the forage fish community models provide information on the potential for species interactions among forage fishes (i.e., intraguild interactions), showing patterns of co‐occurrence across years or tows that are not explained by the species' respective environmental niches. Given the high conditional model fit, especially for the autumn community model, it is possible that the temporal and tow associations identified represent true species interactions. The temporal associations indicate that groups of species are either linked (positive associations) or asynchronous (negative) over time, driven by long‐term processes or behaviors for which our models do not account. The tow‐level associations may indicate species that are spatially associated with each other, such as herring, alewife, and blueback herring, which form large, multispecies schools for foraging (Turner et al., 2016). However, there is a paucity of data on forage fish behavior in the NES ecosystem, and much is still unknown about interspecies interactions within this community. We must exercise caution when inferring species interactions based on residual associations, since they could also indicate that our models are missing environmental covariates that could explain some of this residual covariance. The potential existence of intraguild interactions suggested by our study indicates a need for more research to elucidate these relationships within the forage fish community, especially given evidence of asymmetrical distribution shifts of species in the NES due to climate‐induced warming (Friedland et al., 2020; Hare et al., 2016; Kleisner et al., 2017). A better understanding of intraguild interactions, particularly regarding behaviors influencing the formation and composition of surface FFAs, could allow for more accurate estimations of FFA distribution and prey availability to predators across space and time.

### 4.3 Oceanographic drivers of forage fishes

#### 4.3.1 Community distribution

Submesoscale filaments (FSLE) and front persistence (*Fprob*; autumn: productivity; spring: SST), both dynamic surface features, were the most important environmental drivers for community distribution across seasons, but only for a subset of species. Convergent submesoscale (~10 km) eddy and filament structures aggregate phyto‐ and zooplankton along their ridges or frontal edges (i.e., areas of high FSLE values; d'Ovidio et al., 2010; Smeti et al., 2015), attracting forage fishes to abundant prey resources. Similarly, Suca, Deroba, et al. (2021) found that total kinetic energy, a proxy for mesoscale eddies, was an important driver for the distributions of sand lance, herring, alewife, and mackerel, suggesting, along with our results, that mesoscale and submesoscale eddies are important for the occurrence and abundance of some forage fish species. Filament ridges are also associated with foraging behavior of top predators, including seabirds, sharks, and marine mammals that are likely feeding on forage fishes and other prey that aggregate to these features (Abrahms et al., 2018; Cotté et al., 2011; Della Penna et al., 2015; Kai et al., 2009). Productivity and SST frontal features have also been associated with higher abundance of zooplankton (Genin et al., 2005), forage fishes (Friedland et al., 2020), and top predators (Scales, Miller, Hawkes, et al., 2014), supporting the relationships found in our community models.

Sea surface temperature and depth were important predictors for nearly the entire forage fish community but had a weaker influence than the submesoscale eddies and front persistence. SST is well established as a regulating factor in the distribution of pelagic fishes via physiological thermal niche constraints (Ma et al., 2022), and top predator distributions are known to be associated with SST patterns, as they track prey distributions (Hazen et al., 2013). SST and depth were also consistently predictive of forage fish distributions in single‐species modeling frameworks (Friedland et al., 2020; Holland et al., 2021; Suca, Deroba, et al., 2021). Thus, our models provide further evidence that SST and depth gradients are important for structuring the community distribution of forage fishes.

#### 4.3.2 Aggregating behavior

The complex interplay between MLD and the benthic terrain combined with behavioral mechanisms driven by foraging needs and predator avoidance may be associated with the spatial patterns we found in FFA abundance and size. In the NES, MLD is a temporal indicator of seasonal stratification of the water column with weak stratification and deeper MLD during the winter due to strong upper ocean mixing, contrasting with strong stratification and a shallow MLD during the summer (Cai et al., 2021; Li et al., 2015). Seasonal spatial variation in MLD reflects differences in subsurface temperature and salinity gradients (i.e., thermo‐ and haloclines; collectively, pycnoclines) across the shelf due to localized bathymetry and coastal processes such as freshwater inputs and tidal mixing (Cai et al., 2021; Li et al., 2015). FFA formation is influenced by strong links between seasonal surface stratification, subsurface productivity gradients, and zooplankton distribution with the resources of forage fishes concentrated within a relatively smaller volume of space during the summer and fall, while those resources are dispersed over a larger volume in the winter and spring.

Mixed layer depth was the most important predictor of FFA abundance and the second most important predictor of FFA size. However, the direction of this relationship differed with shallower MLD (stronger stratification) associated with more FFA, and deeper MLD (weaker stratification) associated with larger FFA. Sharp pycnoclines due to strong stratification are associated with high subsurface productivity (Weston et al., 2005), which is, in turn, linked to increased aggregations of zooplankton (Genin, 2004), driving higher abundance of FFA, but smaller individual FFA size. Additionally, links have been found between stratified areas where abrupt changes in topography, such as steep depressions (i.e., negative BPI values), cause internal waves that drive large aggregations of forage fish to the surface (Cox et al., 2018). These conditions are commonly found at the shelf edge or offshore banks, resulting in local upwelling that depresses MLD, allowing resources to disperse and larger FFAs to form.

The regions of highest predicted FFA abundance for our models were also coincident with the relatively shallow depths associated with the mouths of major freshwater inputs, such as the Chesapeake and Delaware Bays, suggesting that in the Mid‐Atlantic and New York Bights, shallow habitat may function both as convergence zone (i.e., a mixing zone between water masses and fine‐scale tidal currents) and refugia from predation (Litz et al., 2014). Moreover, on continental shelves like the NES ecosystem, shallow depth may drive zooplankton aggregation formation by blocking diel migration back to deeper waters (i.e., topographic blockage, Isaacs & Schwartzlose, 1965). Topographic blocking traps those planktonic aggregations in shallower regions, exposing them to forage fish predation, and, subsequently, resulting in FFA formation via trophic focusing (Genin, 2004). However, BPI, a measure of the benthic topography, was a more important predictor than depth for FFA size. In both models, shallower depths were an indication of more and bigger FFA, while larger FFAs were associated with extremely negative BPI, indicating abrupt, benthic depressions or valley bottoms (Lundblad et al., 2006). This connects back to the aforementioned relationship with MLD, where stratified regions interact with abrupt benthic depressions to spur the formation of large FFAs.

#### 4.3.3 Contrasting drivers of distributions and aggregations

While FFA abundance may overlap spatially with community species richness, these patterns are driven by different oceanographic processes. MLD, a subsurface dynamic variable, was important to FFA abundance and size. However, MLD was not important at the community occupancy level with a low effect size in both autumn and spring. In contrast, the environmental drivers with the largest influence on community‐level occupancy were dynamic surface processes: eddies and frontal features. The differences in the oceanographic processes driving community occupancy versus FFA abundance are likely due to the aggregation dataset inherently including behavioral information (i.e., surface schooling behavior), which is absent from the occupancy dataset. Subsurface features describing the vertical water column may be more tightly linked with behavioral processes related to depth (i.e., diel migration, surface aggregation formation). Relatedly, differences in oceanographic drivers may also reflect that FFAs are nested hierarchically within community occupancy, such that FFAs represent finer‐scale structures within the larger community distribution (Fauchald et al., 2000). Conversely, depth, a static habitat feature, was important to all levels of organization (species occupancy, community occupancy, and FFA abundance/size) and has been an important predictor of forage fish abundance in multiple studies of the NES ecosystem (Friedland et al., 2019; Suca, Deroba, et al., 2021). This finding suggests that some static habitat features may influence forage fishes' spatial distribution regardless of scale or may indicate that depth integrates several important processes in one measure.

### 4.4 Implications for ecosystem change

The FFA patterns and community dynamics described by our models are reliant upon relatively recent historical data (FFA: 2012–2019, community SDM: 1997–2019). Due to climate change, the NES is experiencing rapid warming at three times the global average (Pershing et al., 2021) and increased frequency of marine heatwaves (Laufkötter et al., 2020). Concurrent decreases in surface salinity combined with rising temperatures are expected to drive increased seasonal stratification (i.e., MLD; Pershing et al., 2021), which, based on our findings, has the potential to affect the distribution, abundance, and aggregating behavior of forage fishes in this system. Climate‐induced warming has already induced detectable broadscale and seasonal distribution shifts across the trophic web in the NES, from plankton (Chust et al., 2014), fish, and macroinvertebrates (Friedland et al., 2020) to predatory fishes (Muhling et al., 2017) and marine mammals (Pendleton et al., 2022), and is expected to influence the distributions of key forage fishes included in this study, such as sand lance, herring, and menhaden (Hare et al., 2016; Staudinger et al., 2020; Suca, Wiley, et al., 2021). These distribution shifts are not expected to occur symmetrically and may not result in wholescale northward shifts of the present community. Instead, these changes could result in the development of novel, no‐analog communities (i.e., community composition unlike that known from the historical or paleontological record), leading to changes in community relationships among forage fish species and affecting spatiotemporal patterns in aggregating behavior (Williams & Jackson, 2007). Moreover, climate‐induced warming can affect the abundance, size, quality (i.e., lipid content), and intraguild dynamics of forage fishes, disrupting trophic energy transfer to higher trophic level predators and fueling mass mortality events of predators (Arimitsu et al., 2021). Our results highlight the need for additional research into the effects of climate change on subsurface dynamic processes and how those effects may impact trophic interactions.

Additionally, the imminent development of offshore wind energy (i.e., the construction of large‐scale offshore windfarms) in the NES may contribute to meso‐ and submesoscale changes in localized current patterns circulation and subsurface dynamics, such as stratification (Christiansen et al., 2022; Dorrell et al., 2022). Although a recent study shows that wind energy lease areas overlap considerably with the core habitat of forage fish species (Friedland et al., 2023), it is unknown how these habitat alterations may influence forage fish aggregating behavior in the NES (but see Raoux et al., 2017), and, thus, realized prey availability. Unanticipated synergistic interactions between climate change effects, offshore wind energy development, and other anthropogenic stressors, such as pollution and commercial and recreational fisheries, could further alter patterns of forage fish availability across the NES shelf.

### 4.5 Limitations and sources of bias

The NOAA bottom trawl surveys are not designed to sample pelagic or surface schooling species, and there may be differential catchability rates among the forage fishes, as well as biases in size selectivity due to gear design. Moreover, water depth influences the probability of capture of forage fishes by bottom trawls, since midwater forage fishes may be more available to the gear over shallower bottoms (<50 m) compared to deeper waters where they are more likely to be captured only on the deployment and recovery of the gear. However, while these species are defined as pelagic, many do use the entire water column on the continental shelf via diel migration, predator avoidance, foraging, and spawning behaviors (Freon & Misund, 1999). In addition, there was a significant gear change during our study period (2009) that led to notable catch changes for many forage fish species, especially sand lance (Miller et al., 2010). After the gear change, the trawl catch of sand lance has been considered unreliable for the purposes of abundance monitoring (Richardson et al., 2014); however, it has been deemed adequate for presence‐absence occurrence models that span that period (Friedland et al., 2020). Despite bottom trawls not being the ideal sampling method for forage fishes, these surveys are considered to be reliable for measuring abundance for stock assessments and distribution analyses for some forage fish species (Northeast Fisheries Science Center, 2018). To address these issues and control bias, we confined our community analysis to modeling occupancy rather than abundance. In addition, scale is confounded with behavior such that estimating broadscale occupancy in JSDMs rather than finer‐scale abundance may mask behaviorally driven relationships with oceanographic processes, such as community abundance with MLD. Future studies could compare a community JSDM using abundance data to the FFA model results to see if community abundance patterns more closely track FFAs than occupancy; however, species such as sand lance may need to be excluded.

In the aerial survey imagery, submerged fauna may not be detected due to observation conditions (i.e., turbidity, sea state, etc.,), water depth, or seasonal variation in behavior. The digital recorders only capture the top several meters of the water column (Hodgson et al., 2017; Martin Scott, HiDef Aerial Surveying Ltd., pers. comm). Due to variations in vertical distribution patterns of schools, FFAs may be more detectable at shallower depths, while seasonal behaviors of some schooling species, such as shifts to deeper water, may also limit FFA detection by aerial surveys (Freon & Misund, 1999). To address these detection biases, we limited our interpretation to surface FFA patterns, acknowledging that the data does not sample all FFA in the water column. In addition, the aggregation models were limited by the inability to identify FFA composition at species‐level and by the aerial survey sampling frequency, preventing us from integrating the datasets into a combined model for forage fish species and aggregations. Thus, we were reliant upon post‐hoc comparisons of our predicted distributions. Finally, FFA occurrence is a highly ephemeral process, occurring over spatiotemporal scales smaller than the 4 km‐, daily‐scale oceanographic data we used in our models, or the 4–8 aerial surveys/year conducted in this study. Collecting more finer‐scale and local oceanographic and survey data could improve model performance and reveal additional habitat relationships.

## 5 CONCLUSIONS

In the context of rapid climate change and other anthropogenic stressors in the NES, we expect concomitant changes in both the broadscale distribution of forage fishes and the patch‐scale distribution of FFA. Changes in patch‐scale FFA dynamics, as a measure of realized prey availability, are likely to have cascading effects through the food web, impacting predator–prey interactions and driving concurrent changes in predator distributions as they track changing prey availability. Our analysis provides an initial step to better understanding the realized prey availability of upper trophic level predators and how to integrate that information to track current predator–prey interactions and forecast these relationships into an uncertain future. Additionally, our results show that subsurface dynamic processes, such as MLD, are better predictors of FFA than surface features like eddies and fronts, highlighting the need to implement more informative proxies for realized prey availability at the corresponding spatial and organizational scales of predator–prey interactions. When designing marine predator–prey interaction studies, subsurface dynamic variables may be key for detecting these scale‐dependent relationships. Understanding the key drivers of forage fish dynamics at scales relevant to foraging marine predators can aid scientists and managers in implementing effective management and conservation strategies across trophic levels.

## AUTHOR CONTRIBUTIONS

**Chandra Goetsch:** Data curation (equal); formal analysis (lead); methodology (lead); visualization (lead); writing – original draft (lead); writing – review and editing (equal). **Julia Gulka:** Data curation (equal); formal analysis (supporting); writing – review and editing (equal). **Kevin D. Friedland:** Data curation (equal); formal analysis (supporting); methodology (supporting); writing – review and editing (equal). **Arliss J. Winship:** Data curation (equal); methodology (supporting); writing – review and editing (equal). **Jeff Clerc:** Data curation (equal); investigation (equal); writing – review and editing (equal). **Andrew Gilbert:** Data curation (equal); investigation (equal); writing – review and editing (equal). **Holly F. Goyert:** Methodology (supporting); writing – review and editing (equal). **Iain J. Stenhouse:** Data curation (equal); investigation (equal); writing – review and editing (equal). **Kathryn A. Williams:** Data curation (equal); funding acquisition (supporting); investigation (equal); writing – review and editing (equal). **Julia R. Willmott:** Data curation (equal); investigation (equal); writing – review and editing (equal). **Melinda L. Rekdahl:** Writing – review and editing (equal). **Howard C. Rosenbaum:** Writing – review and editing (equal). **Evan M. Adams:** Conceptualization (lead); data curation (equal); formal analysis (lead); funding acquisition (lead); investigation (equal); methodology (lead); visualization (supporting); writing – original draft (supporting); writing – review and editing (equal).

## CONFLICT OF INTEREST STATEMENT

The authors declare no conflict of interest.

## Data Availability

NOAA bottom trawl data (NOAA InPort Catalog IDs: 22560 and 22,561) are available at https://www.fisheries.noaa.gov/inport. Aerial digital survey data for the New York Project (OBIS‐SEAMAP IDs: 1817, 1818, 1994, and 2073) are available at http://seamap.env.duke.edu. Aerial digital survey data for the Mid‐Atlantic Project (Catalog IDs: 115, 148, 168) are available from NOAA NCCOS Northwest Atlantic Seabird Catalog (v.0.6.2) upon request. A complete list of the publicly available datasets used for this manuscript, including query details, can be found in Appendix 1: Section 1 and Appendix 1: Tables A1 and A2. Example code for the NIMBLE FFA models is available at https://github.com/cgoetsch/Forage_Fish_Aggregation_Models.

## APPENDIX 1

### DATA INFORMATION

#### Section 1: Response data accessibility information

##### Bottom trawl data

Northeast Fisheries Science Center, 2020: Fall Bottom Trawl Survey from 1997 to 2019, Catalog ID: 22560. NOAA National Centers for Environmental Information, https://www.fisheries.noaa.gov/inport/item/22560.

Northeast Fisheries Science Center, 2020: Spring Bottom Trawl Survey from 1998 to 2019, Catalog ID: 22561. NOAA National Centers for Environmental Information, https://www.fisheries.noaa.gov/inport/item/22561.

##### Aerial digital survey data

APEM and Normandeau Associates prepared for New York State Energy Research and Development Authority. 2018. Digital Aerial Baseline Survey of Marine Wildlife in Support of Offshore Wind Energy – OPA 2016. OBIS‐SEAMAP ID: 1817. Data accessible from OBIS‐SEAMAP (http://seamap.env.duke.edu/dataset/1817).

APEM and Normandeau Associates prepared for New York State Energy Research and Development Authority. 2018. Digital Aerial Baseline Survey of Marine Wildlife in Support of Offshore Wind Energy – WEA 2016. OBIS‐SEAMAP ID: 1818. Data accessible from OBIS‐SEAMAP (http://seamap.env.duke.edu/dataset/1818).

APEM and Normandeau Associates prepared for New York State Energy Research and Development Authority. 2019. Digital Aerial Baseline Survey of Marine Wildlife in Support of Offshore Wind Energy – OPA 2017. OBIS‐SEAMAP ID: 1994. Data accessible from OBIS‐SEAMAP (https://seamap.env.duke.edu/dataset/1994).

APEM and Normandeau Associates prepared for New York State Energy Research and Development Authority. 2019. Digital Aerial Baseline Survey of Marine Wildlife in Support of Offshore Wind Energy – OPA 2018. OBIS‐SEAMAP ID: 2073. Data accessible from OBIS‐SEAMAP (https://seamap.env.duke.edu/dataset/2073).

Biodiversity Research Institute and HiDef Aerial Surveying prepared for the Department of Energy Mid‐Atlantic 2015. Mid‐Atlantic Digital Aerial Survey 2012 – DOE/BRI, ID 115. Accessible through the NOAA National Centers for Coastal Ocean Science (NCCOS), Northwest Atlantic Seabird Catalog Version 0.6.2. Data available upon request: Contact Arliss Winship, CSS, Inc. under contract to NOAA, Biogeography Branch, Marine Spatial Ecology Division, NCCOS, 1305 East–West Hwy, SSMC‐4, N/SCI‐1, #9245, Silver Spring, MD 20910, arliss.winship@noaa.gov. Data can also be downloaded at https://briwildlife.org/wp‐content/uploads/2021/09/BRI_DOE_Hidef_aerialSurveys_final.zip.

Biodiversity Research Institute and HiDef Aerial Surveying prepared for the Department of Energy. 2015. Mid‐Atlantic Digital Aerial Survey 2013 – DOE/BRI, ID 148. Accessible through the NOAA National Centers for Coastal Ocean Science (NCCOS), Northwest Atlantic Seabird Catalog Version 0.6.2. Data available upon request: Contact Arliss Winship, CSS, Inc. under contract to NOAA, Biogeography Branch, Marine Spatial Ecology Division, NCCOS, 1305 East–West Hwy, SSMC‐4, N/SCI‐1, #9245, Silver Spring, MD 20910, arliss.winship@noaa.gov. Data can also be downloaded at https://briwildlife.org/wp‐content/uploads/2021/09/BRI_DOE_Hidef_aerialSurveys_final.zip.

Biodiversity Research Institute and HiDef Aerial Surveying prepared for the Department of Energy. 2015. Mid‐Atlantic Digital Aerial Survey 2014 – DOE/BRI, ID 168. Accessible through the NOAA National Centers for Coastal Ocean Science (NCCOS), Northwest Atlantic Seabird Catalog Version 0.6.2. Data available upon request: Contact Arliss Winship, CSS, Inc. under contract to NOAA, Biogeography Branch, Marine Spatial Ecology Division, NCCOS, 1305 East–West Hwy, SSMC‐4, N/SCI‐1, #9245, Silver Spring, MD 20910, arliss.winship@noaa.gov. Data can also be downloaded at https://briwildlife.org/wp‐content/uploads/2021/09/BRI_DOE_Hidef_aerialSurveys_final.zip.

#### Section 2: Front detection

We detected sea surface temperature (SST) and chlorophyll *a* (Chl*a*) fronts from daily rasters (Appendix 1 Table A2), using the Cayula‐Cornillon Fronts tool in the Marine Geospatial Ecology (MGET) toolbox (version 0.8a75, Roberts et al., 2010) for ArcGIS (version 10.8.1, ESRI Inc.). This tool uses the Cayula and Cornillon SIED (Single Image Edge Detection) algorithm to identify fronts (Cayula & Cornillon, 1992). For the detection of SST fronts, we used a 0.4°C temperature threshold (Cayula & Cornillon, 1992). To increase detection of coastal and smaller scale fronts, we adjusted the default tool settings to a 16 × 16 pixel window, a 4 window stride, and a 5 × 5 kernel (Roa‐Pascuali et al., 2015). We also adjusted the spatial cohesion parameters to reflect the smaller histogram window: 0.87 minimum single population spatial cohesion and 0.88 minimum global population spatial cohesion (Cayula & Cornillon, 1992). For the detection of Chl*a* fronts, we optimized the CCA parameters to better detect coastal fronts, using a 0.4 mg/m^3^ threshold. As with the SST fronts, we adjusted the parameters as follows: a 16 × 16 pixel window, a 3 window stride and a 5 × 5 kernel, changing the minimum single and global population cohesion values accordingly. We also set the minimum criterion function to 0.74 to allow “curvier” fronts to be identified (Cayula & Cornillon, 1992). We used the thin option to ensure one‐pixel‐width fronts.

Frontal gradients were calculated for SST and Chl*a* using the Belkin O'Reilly gradient algorithm (Belkin & O'Reilly, 2009) with the detectFronts function in the grec package in R (version 1.4.1). Then, all detected fronts and calculated gradients over a 7‐day moving window were combined into daily composite frontal maps (Scales, Miller, Embling, et al., 2014), and used to calculate two frontal metrics: *Fprob* and *Fmean* (Miller, 2009; Suberg et al., 2019) for each day of the study period (1997–2019). *Fprob* is a measure of front persistence and is calculated as the probability of a front being detected each day over the rolling 7‐day window. *Fmean* is a measure of front intensity and is calculated as the average of the frontal gradient within the detected fronts.

**TABLE A1:** Static environmental data details and access information.

Covariate	Description	Spatial resolution	Data source	References
BPI	Benthic position index: derived from slope.	350 m	Derived from NAMERA^a^ bathymetry data with an inner radius = 5 and outer radius = 50	Lundblad et al. (2006)
Complexity	Terrain ruggedness index	500 m	Derived from NOAA Coastal Relief Model, NCEI https://doi.org/10.7289/V5MS3QNZ	Riley et al. (1999)
Depth (log)	Log of the bathymetric elevation	15 arc‐sec	General bathymetric chart of the oceans (GEBCO): https://www.gebco.net/ https://doi.org/10.5285/a29c5465‐b138‐234d‐e053‐6c86abc040b9	GEBCO Compilation Group (2020)
Distance to shelf	Distance in kilometers to the 200 m isobath representing the continental shelf break	4 km	Derived from GSHHG^b^ (Global Self‐consistent Hierarchical High Resolution Geography) Shorelines Version 2.3.7, 2017, using ArcGIS 10.8.1.	Wessel and Smith (1996)
Distance to shore	Distance in kilometers to the GSSHHG shoreline	4 km	Derived from GSHHG^b^ (Global Self‐consistent Hierarchical High Resolution Geography) Shorelines Version 2.3.7, 2017, using ArcGIS 10.8.1.	Wessel and Smith (1996)
Planform curvature	Benthic planform curvature	15 arc‐sec	Derived from Gaussian smoothed (2 km spatial scale) GEBCO bathymetry	Winship et al. (2018)
Profile curvature	Benthic profile curvature	15 arc‐sec	Derived from Gaussian smoothed (2 km spatial scale) GEBCO bathymetry	Winship et al. (2018)
Rugosity	Variation in amplitude of the height of the bathymetric terrain as given by the ratio of the actual to the geometric surface area	500 m	Derived from NOAA Coastal Relief Model, NCEI https://doi.org/10.7289/V5MS3QNZ	Friedman et al. (2012)
Seabedforms	Categorical seabed topography; combination of the seabed topographical position and shape	80 m	North Atlantic Marine Ecoregional Assessment (NAMERA)^a^	Greene et al. (2010)
Slope	Benthic slope	15 arc‐sec	Derived from Gaussian smoothed (2 km scale) GEBCO bathymetry, using DEM Surface Tools^c^	Winship et al. (2018)
Slope of slope	Slope of the benthic slope	15 arc‐sec	Derived from Gaussian smoothed (2 km scale) GEBCO bathymetry, using DEM Surface Tools^c^	Winship et al. (2018)
Sediment	Benthic soft sediment grain size (mm)	700 m	North Atlantic Marine Ecoregional Assessment (NAMERA)^a^	Greene et al. (2010)
VRM	Vector ruggedness measure; the variation in the 3‐d orientation of cells in a neighborhood	350 m	Derived from NAMERA^a^ bathymetry data	Sappington et al. (2007)

^a^ Data access: http://www.conservationgateway.org/ConservationByGeography/NorthAmerica/UnitedStates/edc/reportsdata/marine/namera/namera/Pages/Spatial‐Data.aspx.

^b^ Data access: https://www.ngdc.noaa.gov/mgg/shorelines/.

^c^ Data access: Jenness, 2013.

**TABLE A2:** Dynamic environmental data details.

Covariate	Description	Type	Spatial resolution	Data source	Reference
BottomT	Bottom temperature; potential temperature at the sea floor	Subsurface	8 km	GLORYS12V1 global ocean eddy‐resolving model: https://doi.org/10.48670/moi‐00021	Lellouche et al. (2021)
Chl*a*	Chlorophyll‐*a* concentration (mg/m^3^)	Surface	4 km	Copernicus GlobColour interpolated cloud‐free product: https://doi.org/10.48670/moi‐00100	Garnesson et al. (2019)
Chl *Fmean*	7‐day composite mean Chl*a* front gradient	Surface	4 km	Derived from Copernicus GlobColour chlorophyll‐*a*	Miller (2009) and Suberg et al. (2019)
Chl *Fprob*	7‐day composite probability of observing a Chl*a* front	Surface	4 km	Derived from Copernicus GlobColour chlorophyll‐*a*	Miller (2009) and Suberg et al. (2019)
Currents	Surface current velocities (ms^−1^): u (eastward) and v (northward) velocities	Surface	8 km	GLORYS12V1 global ocean eddy‐resolving model: https://doi.org/10.48670/moi‐00021	Lellouche et al. (2021)
Eddies	Altimetry‐derived mesoscale cyclonic/anticyclonic eddies	Surface	‐	Altimetric Mesoscale Eddy Trajectories Atlas 2.0 distributed by AVISO^a^	Chelton et al. (2011)
FSLE	Finite‐size Lyapunov exponents; identifies Lagrangian coherent structures known as submesoscale filaments	Surface	4 km	Altimeter products produced by Ssalto/Duacs with Locean and CTOH and distributed by Aviso+, with support from CNES; https://doi.org/10.24400/527896/a01‐2022.002	d'Ovidio et al. (2004)
MLD	Mixed layer depth defined by the change in water density (sigma *t*)	Subsurface	8 km	GLORYS12V1 global ocean eddy‐resolving model: https://doi.org/10.48670/moi‐00021	Lellouche et al. (2021)
Salinity	Sea surface salinity	Surface	8 km	GLORYS12V1 global ocean eddy‐resolving model: https://doi.org/10.48670/moi‐00021	Lellouche et al. (2021)
SSHA	Sea surface height anomaly (sea level anomaly) with respect to the 20‐year mean	Surface	0.25 deg	Salto/Duacs altimetry product distributed by CMEMS: https://doi.org/10.48670/moi‐00148	Taburet et al. (2019)
SST	Sea surface temperature	Surface	4 km	OSTIA global gap‐free reprocessed product: https://doi.org/10.48670/moi‐00168	Good et al. (2020)
SST anomaly	SST Seasonal anomaly: difference from the seasonal SST mean of the study area	Surface	4 km	Derived from OSTIA SST	‐
SST *Fmean*	7‐day composite mean SST front gradient	Surface	4 km	Derived from OSTIA SST	Miller (2009) and Suberg et al. (2019)
SST *Fprob*	7‐day composite probability of observing an SST front	Surface	4 km	Derived from OSTIA SST	Miller (2009) and Suberg et al. (2019)
Turbidity	Diffuse attenuation coefficient at 490 (m^−1^) of the downwelling irradiant at 490 nm (KD 490)	Surface	4 km	Copernicus GlobColour interpolated cloud‐free product: https://doi.org/10.48670/moi‐00106	Sathyendranath et al. (2019)

*Note*: All covariates were available at a daily temporal resolution.

^a^ The altimetric Mesoscale Eddy Trajectories Atlas (META2.0) was produced by SSALTO/DUACS and distributed by AVISO+ with support from CNES, in collaboration with Oregon State University with support from NASA. https://www.aviso.altimetry.fr/en/data/products/value‐added‐products/global‐mesoscale‐eddy‐trajectory‐product.html.

## APPENDIX 2

### FORAGE FISH MODELS

**TABLE A1:** Markov Chain Monte Carlo (MCMC) posterior distribution sampling parameters for the forage fish community models.

Model	Total iterations (million)	Burn‐in (million)	Thin	Samples per chain	Total samples (4 chains)
Autumn *Fprob*	2.5	1	750	2000	8000
Autumn *Fmean*	3.3	1.5	900	2000	8000
Spring *Fprob*	3.6	1.5	700	3000	12,000
Spring *Fmean*	3.6	1.5	700	3000	12,000

*Note*: Models were run with different sampling parameters to achieve adequate chain convergence and mixing.

**TABLE A2:** Beta parameter estimates for (a) autumn and (b) spring community models.

Species	Depth (log)	Rugosity	Sediment	SST	Chla	FSLE	MLD	Salinity	SST *Fprob*	Chl *Fprob*
**(a)**										
alewif	−0.17	0.26	−0.68	−0.56	0.11	5.27	−0.01	−0.37	0.36	−1.06
atherr	−1.57	−0.75	−0.22	0.47	−0.04	−0.32	0.06	0.07	0.78	3.21
atlher	0.58	0.09	−0.16	−0.28	0.03	3.77	0.01	−0.46	−0.08	0.53
atlmac	−0.50	0.07	−0.03	−0.25	0.02	−0.93	0.00	0.04	0.31	−0.73
atlmen	−0.76	−0.09	−0.21	0.00	0.03	3.52	−0.01	−0.10	−0.84	−0.46
atsaur	0.14	−0.04	0.12	−0.08	0.02	−2.08	0.00	−0.22	0.27	0.56
bayanc	−1.43	−0.01	−0.12	0.15	0.09	2.17	0.01	−0.30	−0.75	0.51
bluher	−0.97	0.03	−0.59	−0.55	0.09	2.57	−0.02	−0.25	0.39	−0.67
butter	−0.69	−0.08	−0.23	0.03	0.04	−0.59	0.01	−0.08	0.08	−0.07
rherri	−0.42	−0.12	−0.10	0.09	−0.04	−0.29	−0.03	−0.04	−0.55	0.53
sandla	−0.32	−0.19	0.33	−0.07	−0.09	−0.66	−0.01	0.00	−0.11	0.53
silanc	−0.67	−0.15	−0.11	0.03	0.03	−1.49	−0.01	0.09	0.11	−0.50
spsard	−1.03	−0.43	−0.25	0.35	−0.05	−0.73	0.05	0.13	0.13	0.40
stranc	−1.89	−0.81	−0.35	0.47	0.01	1.71	0.07	0.07	−0.43	3.63
Absolute mean	0.80	0.22	0.25	0.24	0.05	1.87	0.02	0.16	0.37	0.96
**(b)**										
alewif	0.55	‐	−0.35	−0.16	0.15	7.69	−0.02	−0.31	−0.16	−0.39
atlher	0.02	‐	−0.12	−0.15	0.07	4.85	0.00	−0.22	−0.61	0.52
atlmac	−0.18	‐	−0.21	−0.17	−0.17	2.21	0.03	0.41	−1.04	−0.27
atlmen	−0.58	‐	−0.41	0.07	−0.03	−0.31	−0.01	−0.05	−1.86	0.94
atlsil	−0.88	‐	−0.23	−0.36	0.00	−0.43	−0.04	−0.07	1.06	−0.32
bayanc	−0.72	‐	−0.31	0.16	−0.05	0.04	0.01	−0.21	−0.87	−0.42
bluher	−0.17	‐	−0.45	−0.06	0.06	10.22	−0.03	−0.27	−0.67	−0.47
butter	0.04	‐	−0.36	0.15	−0.06	−2.69	0.00	0.30	−0.11	−0.64
sandla	−0.55	‐	0.27	−0.14	0.04	3.19	0.00	0.16	0.34	0.44
stranc	−0.73	‐	−0.60	0.12	−0.08	3.29	0.00	0.21	−0.17	−0.40
Absolute mean	0.44	‐	0.33	0.15	0.07	3.49	0.01	0.22	0.69	0.48

*Note*: Values in black are supported with at least 0.95 posterior probability; grayed‐out values had < 0.95 posterior probability. Species codes are defined in Table 1.

**TABLE A3:** Estimated prevalence (mean probability of occurrence) of species within distinct forage fish communities for the (a) autumn and (b) spring community models.

Species	Type 1	Type 2	Type 3	Type 4	Type 5	Type 6
**(a)**						
alewif	0.315	0.637	0.200	0.118	0.007	0.000
atherr	0.286	0.000	0.000	0.000	0.002	0.000
atlher	0.051	0.845	0.736	0.334	0.072	0.138
atlmac	0.546	0.219	0.094	0.204	0.055	0.005
atlmen	0.629	0.003	0.000	0.013	0.023	0.000
atsaur	0.020	0.070	0.064	0.045	0.017	0.014
bayanc	0.917	0.000	0.000	0.007	0.033	0.000
bluher	0.649	0.086	0.003	0.040	0.004	0.000
butter	0.998	0.525	0.388	0.758	0.789	0.129
rherri	0.196	0.000	0.000	0.003	0.015	0.000
sandla	0.160	0.020	0.020	0.056	0.037	0.006
silanc	0.311	0.000	0.000	0.002	0.007	0.000
spsard	0.117	0.000	0.000	0.000	0.002	0.000
stranc	0.590	0.000	0.000	0.000	0.011	0.000
**(b)**						
alewif	0.237	0.654	0.581	0.252	0.162	0.164
atlher	0.424	0.402	0.282	0.266	0.134	0.027
atlmac	0.031	0.125	0.174	0.118	0.108	0.074
atlmen	0.374	0.003	0.001	0.017	0.014	0.008
atlsil	0.311	0.003	0.000	0.006	0.000	0.000
bayanc	0.654	0.004	0.000	0.029	0.023	0.019
bluher	0.712	0.176	0.081	0.155	0.083	0.007
butter	0.114	0.163	0.327	0.217	0.479	0.858
sandla	0.308	0.030	0.011	0.084	0.021	0.002
stranc	0.208	0.000	0.000	0.004	0.006	0.010

*Note*: Species codes are defined in Table 1.
